# Exploring novel bacterial terpene synthases

**DOI:** 10.1371/journal.pone.0232220

**Published:** 2020-04-30

**Authors:** Gajendar Komati Reddy, Nicole G. H. Leferink, Maiko Umemura, Syed T. Ahmed, Rainer Breitling, Nigel S. Scrutton, Eriko Takano

**Affiliations:** 1 Manchester Synthetic Biology Research Centre SYNBIOCHEM, Manchester Institute of Biotechnology, School of Chemistry, University of Manchester, Manchester, England, United Kingdom; 2 Future Biomanfacturing Research Hub (FBRH), Manchester Institute of Biotechnology, School of Chemistry, University of Manchester, Manchester, England, United Kingdom; 3 Bioproduction Research Institute, National Institute of Advanced Industrial Science and Technology (AIST), Tsukuba, Ibaraki, Japan; 4 AIST-Waseda University Computational Bio Big-Data Open Innovation Laboratory (CBBD-OIL), AIST, Tsukuba, Ibaraki, Japan; CSIR-Central Institute of Medicinal and Aromatic Plants, INDIA

## Abstract

Terpenes are the largest class of natural products with extensive structural diversity and are widely used as pharmaceuticals, herbicides, flavourings, fragrances, and biofuels. While they have mostly been isolated from plants and fungi, the availability and analysis of bacterial genome sequence data indicates that bacteria also possess many putative terpene synthase genes. In this study, we further explore this potential for terpene synthase activity in bacteria. Twenty two potential class I terpene synthase genes (TSs) were selected to represent the full sequence diversity of bacterial synthase candidates and recombinantly expressed in *E*. *coli*. Terpene synthase activity was detected for 15 of these enzymes, and included mono-, sesqui- and diterpene synthase activities. A number of confirmed sesquiterpene synthases also exhibited promiscuous monoterpene synthase activity, suggesting that bacteria are potentially a richer source of monoterpene synthase activity then previously assumed. Several terpenoid products not previously detected in bacteria were identified, including aromandendrene, acora-3,7(14)-diene and longiborneol. Overall, we have identified promiscuous terpene synthases in bacteria and demonstrated that terpene synthases with substrate promiscuity are widely distributed in nature, forming a rich resource for engineering terpene biosynthetic pathways for biotechnology.

## Introduction

Terpenoids, or isoprenoids, are a large class of structurally diverse natural products, with more than 80,000 compounds described in the Dictionary of Natural Compounds (http://dnp.chemnetbase.com). The vast majority of terpenoids have been isolated from plants and fungi; however, bacteria are also known producers of volatile odoriferous metabolites. All terpenoids are synthesised from the universal C5 isoprenoid precursors isopentenyl diphosphate (IPP) and dimethylallyl diphosphate (DMAPP), which are joined by isoprenyl transferases to form isoprenyl diphosphate substrates of varying lengths, such as geranyl diphosphate (GPP, C10), farnesyl diphosphate (FPP, C15) and geranylgeranyl diphosphate (GGPP, C20). Terpene synthases (TSs) convert the linear isoprenyl diphosphate substrates into structurally diverse mono- (C10), sesqui- (C15) and diterpene (C20) scaffolds. Due to their structural diversity, terpenoids have a wide range of industrial applications as pharmaceuticals, flavourings, fragrances, antimicrobials, pesticides and alternative fuels [1]. Recovery of terpenoids from natural sources is hampered by the accumulation of low quantities of target compounds and the dependence on crop yields. Likewise, chemical synthesis is hampered by the structural complexity of terpenoids, which often contain multiple stereo-centres, making synthetic chemistry expensive and environmentally costly. Recent advances in synthetic biology offer new routes to the diverse terpenoid chemistry via the expression of TSs in the presence of heterologous isoprenoid production pathways in engineered microorganisms [2–7].

TSs are capable of creating chemical diversity by converting linear precursors, via diphosphate abstraction (class I enzymes) or protonation of an olefinic double bond (class II), resulting in highly reactive allylic cations. The allylic cations can then undergo wider chemical reactions such as intramolecular attack on olefinic double bonds, hydride or proton migration, and Wagner–Meerwein rearrangement. Ultimately, the reaction is terminated by deprotonation or nucleophilic attack, furthermore, the initial product may be re-ionised for a second round of processing [8]. Hydrophobic residues form a pocket which protects the cationic intermediates from the undirected attack of water, and aromatic residues stabilize charged intermediates by cation–π interactions [9,10]. Such relatively inert active sites result in very little overall sequence similarity between TSs. Despite this overall low sequence similarity, TSs from plants, fungi, and bacteria, share a common fold and contain highly conserved metal-binding motifs [11]. Class I enzymes exhibit a highly conserved aspartate-rich motif (DDXXD) at the active centre and a (N,D)D(L,I,V)X(S,T)XXXE consensus sequence (NSE/DTE triad) involved in Mg^2+^ cofactor binding that complexes the diphosphate for ionization in order to cyclise the linear precursors. Similarly, Class II enzymes share a highly conserved DXDD motif for protonation of the substrate (11). Interestingly, a newly discovered class of microbial-like TPSs (MTPSL) from plants, alongside the common class I DDXXD signature motif, contain additional DDXXXD and DDXXX motifs suggesting a divergent evolutionary origin [12–14].

Actinomycetes, including many *Streptomyces* species, are known producers of terpenoids, including the terpene derivatives responsible for the characteristic musty odours of moist soil, geosmin and 2-methylisoborneol [15,16]. Geosmin synthase is an unusual bi-domain TS which exhibits an atypical mechanism, the N-terminal domain catalyses the cyclisation of FPP to germacradienol, while the C-terminal domain catalyses the conversion of germacradienol to geosmin resulting in the elimination of acetone [17]. Pentalenolactone is a sesquiterpenoid antibiotic that has been isolated from over 30 different *Streptomyces* species [18–20], and pentalenene synthase, the enzyme responsible for the cyclisation of FPP into pentalenene (the pentalenolactone hydrocarbon precursor), which is the first characterised bacterial TS [21]. After geosmin and 2-methylisoborneol synthases, epi-isozizaene synthases are the most widespread TSs found in bacteria, invariably from *Streptomyces* species [22]. Most characterised bacterial TSs exhibit sesquiterpene synthase activity, and only a few true bacterial monoterpene synthases are known so far [6,23].

Recent genome mining studies suggest that bacteria are a potentially much richer source of TSs, including enzymes from Gram-negative bacteria such as Cyanobacteria and Proteobacteria [22,24]. Since bacterial TSs, in particular, show only weak sequence conservation, an amino acid sequence-based product prediction for TSs is not possible. Hidden Markov Model (HMM) profiles are more suitable for sensitive database searches as they use statistical descriptions of a consensus sequence from one family. This approach was first applied to bacterial TSs by Komatsu et al (2008) to identify 2-methylisoborneol synthases in Actinomycetes [25]. Using similar approaches, the number of uncharacterised bacterial terpene cyclases has recently increased to more than 600 regular class I terpene synthases, 400 geosmin synthases and over 120 2-methylisoborneol synthases, due to the ever-increasing availability of genome sequencing data [22,24,26–28].

In this study, we further explore the potential for terpene synthase activity in bacteria. We have constructed a neighbour-joining tree containing 2,167 putative bacterial terpene cyclases/synthases, including geosmin synthases, 2-methylisoborneol synthases, and regular TSs, by using the Pfam HMM motif containing the two signature domains of the class I terpene synthase for the analysis of publicly available bacterial genome sequences. We were especially interested in determining the biochemical function of presumptive TSs that are separated by the neighbour-joining analysis into isolated clusters that do not contain any other assigned function and belong to diverse bacterial species other than *Streptomyces*. We have selected 22 potential class I TSs from different clusters and a diverse range of bacterial species for e.g. thermophilic or thermotolerant Actinobacteria (Actinomycetes), Proteobacteria, Firmicutes, Flavobacteria and Myxobacteria for recombinant expression in *Escherichia coli*. We screened for potential TS activity with GPP, FPP, and GGPP substrates using a combination of *in vitro* assays on purified recombinant proteins, and *in vivo* assays employing an engineered *E*. *coli* strain containing a heterologous mevalonate (MVA) pathway [7,29]. TS activity was detected for 15 enzymes, and included mono-, sesqui- and di-terpene synthase activities. Active TSs was obtained from several Proteobacteria species, as well as radio- or thermo-tolerant Actinobacteria species. Interestingly, several enzymes were active on more than one prenyl-pyrophosphate substrate. In particular, a number of sesquiterpene synthases also exhibited monoterpene synthase activity, suggesting that bacteria are a potentially much richer source of monoterpene synthase activity then previously assumed. Several new terpenoid products not seen before in bacterial species were detected, including aromandendrene, acora-3,7(14)-diene and longiborneol.

## Materials and methods

### Bioinformatic screening and selection of terpene synthases

**Fig 1:**
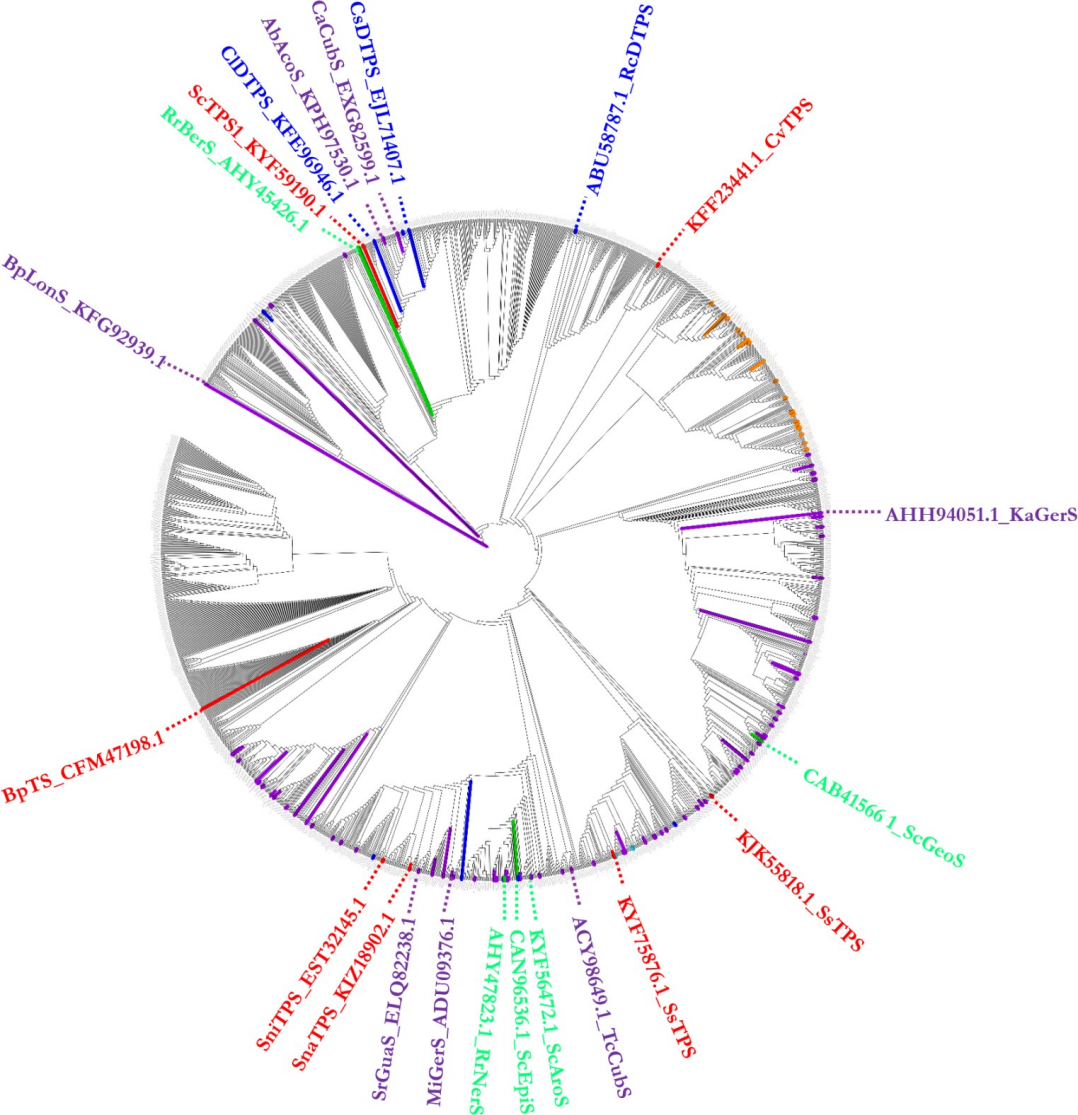
Neighbor-joining analysis of bacterial TSs. Twenty two TSs selected in this study are named. TSs in red are those whose functionality could not be characterized in this work. The functionality of the selected TSs characterized in this study is indicated in colour: they produce either sesquiterpenoids (purple), diterpenoids (blue), or a mixture of sesqui- and mono-terpenoids (green). Known TSs were annotated in the tree and branches were indicated in colour based on their functionality, monoterpenoids (light blue), 2- methyisoborneol (orange), sesquiterpenoids (purple) and diterpenoids (blue) and also listed in S4 Table.

Based on previous work [30], the HMM motif of Terpene_synth_C, which contains the two signature domains of class I TSs (DDXXD and (N,D)D(L,I,V)X(S,T)XXXE), with the HMM score larger than 23 was searched against 73,714 total protein sequences from 8,509 complete genomes of bacteria in the NCBI database (https://www.ncbi.nlm.nih.gov/genome/, 20 June, 2016), using the HMM search module in HMMER ver. 3.1b2 (hmmer.org). From the hits, a neighbour-joining tree of 2,167 known and unknown mono-, sesqui- and di-TSs was generated using the MAFFT program, ver.7.299 [31] with the options of—tree out—global pair—reorder—distout, and visualized on the iTOL (https://itol.embl.de/) [32] (Fig 1).

### Plasmid construction

For *in vivo* diterpenoid synthase activity, 8 TSs listed in Table 1 were amplified by pETM11-fw and pETM11-rev using a pETM11-TS as a template containing homologous sequences at both ends for Gibson assembly [33]. *IspA*M22 (D2G, C155G) inserts were created by amplification of the *ispA* gene from *E*. *coli* DH5α with primers containing the desired base changes (S1 Table). A primer extension method was used to amplify the full-length gene which was inserted into pBbA2k-ispAM22-TS (for full list see S1 Table) by annealing at 50°C using Gibson assembly mix (NEB). For mono- or sesqui-terpene production pBbb2a-GPPS plasmid backbone was used for cloning and TSs (S1 Table) were amplified by pETM11-fw and pETM11-rev using a pETM11-TS as a template containing homologous sequences at both ends for Gibson assembly.

**Table 1:** List of 22 tested TSs and their activity.

	Gene ID		Source	Products		
				GPP (C_10_)	FPP (C_15_)	GGPP(C_20_)
**1**	AHY47823.1	RrNerS	*Rubrobacter radiotolerans*	β-Myrcene‡, Ocimene†, R/S-Linalool†, Geraniol‡	(±)-Nerolidol#, α/β-Farnesene‡, Farnesol‡	NT
**2**	AHY45426.1	RrBerS	*Rubrobacter radiotolerans*	β-Myrcene‡, Linalool#, trans-Geraniol‡	α-Bergamotene#, (±)-trans Farnesene‡, (±)-trans Nerolidol‡, Farnesol‡	NT
**3**	KYF56472.1	ScAroS	*Sorangium cellulosum*	cis and trans- Ocimene‡, β-Pinene‡, R/S-Linalool‡	Aromandendrene#	NT
**4**	KFG92939.1	BpLonS	*Burkholderia paludis*	Geraniol‡	(±)-Cadinene#, Longiborneol#	NT
**5**	AHH94051.1	KaGerS	*Kutzneria albida DSM 43870*	ND	Germacrene D# Germacradienol#,	ND
**6**	KPH97530.1	AbAcoS	*Actinobacteria bacterium OV450*	ND	Copaene†, Acora-3,7(14)-diene†	NT
**7**	ADU09376.1	MiGerS	*Micromonospora sp*. *L5*	ND	Germacrene A#, 1(10),4,11-Germacratriene#	NT
**8**	CAN96536.1	ScEpiS	*Sorangium cellulosum So ce56* [49]	cis and trans- Ocimene#, R/S Linalool#, β-Pinene	10-epi-α/β-Cubinene#, β-Copaene #, Cadina-3,5-diene#, Cubebol#, α/γ/ δ-Cadinene#, 1,10-di-epi-Cubebol#, Cubenol#	ND
**9**	CAB41566.1	ScGeoS	*Streptomyces coelicolor* A3(2)	β-Myrcene†, β-Ocimene†, Linalool†, Geraniol†	Geosmin†, Germacradienol#, Germacrene D#	NT
**10**	ACY98649.1	TcCubS	*Thermomonospora curvata* DSM 43183	ND	Cubebol‡	ND
**11**	EXG82599.1	CaCubS	*Cryptosporangium arvum* DSM 44712	ND	Cubebol‡	ND
**12**	ELQ82238.1	SrGuaS	*Streptomyces rimosus*	ND	Guaia-1(10),11-diene‡, (+)-γ-Gurjunene‡	ND
**13**	ABU58787.1	RcDTPS	*Roseiflexus castenholzii* DSM 13941	ND	ND	2–3 unidentified compounds‡
**14**	EJL71407.1	CsDTPS	*Chryseobacterium sp*. *CF314*	ND	ND	2 unidentified compounds‡
**15**	KFE96946.1	ClDTPS	*Chryseobacterium*. *Luteum*	ND	ND	2–3 unidentified compounds‡
**16**	CFM47198.1	BpTPS	*Burkholderia pseudomallei*	ND	ND	ND
**17**	KYF59190.1	ScTPS1	*Sporangium cellulosum*	ND	ND	ND
**18**	KFF23441.1	CvTPS	*Chryseobacterium vrystaatense*	ND	ND	ND
**19**	KJK55818.1	SsTPS	*Saccharothrix sp*. *ST-888*	ND	ND	ND
**20**	KYF75876.1	ScTPS2	*Sporangium cellulosum*	ND	ND	ND
**21**	EST32145.1	SniTPS	*Streptomyces niveus* NCIMB 11891	ND	ND	ND
**22**	KIZ18902.1	SnaTPS	*Streptomyces natalensis* ATCC 27448	ND	ND	ND

The products obtained by *in vitro* assay with purified TSs upon incubation with GPP and FPP or *in vivo* cultivation in *E*. *coli* are listed.

†–produced in *in vitro* conditions

‡–produced in *in vivo* conditions

#—produced in *in vitro* and *in vivo* conditions; ND–not detected, NT–not tested

Genes encoding selected TS from different bacteria were codon optimised for expression in *E*. *coli* (S1 Table), synthesized, and sub-cloned into pETM11 with N-terminal His-tag (GeneArt, Life Technologies). Protein sequence of the selected TSs are shown S2 Table.

### Bacterial strains and growth conditions

For *in vitro* analysis, pre-cultures, LB medium (10 g/L tryptone, 5 g/L yeast extract, 5 g/L NaCl; pH 7.0) were used and cultures were incubated at 37°C for overnight (containing kanamycin at 50 mg/L) in 5 mL. For protein expression, precultures were diluted (1/1000) in 5 mL 2xYT medium (16 g/L Tryptone, 10 g/L Yeast Extract, 5.0 g/L NaCl) and 50 μL was inoculated into Auto Induction Medium Terrific Broth (AIMTB, Formedium) and incubated at 37°C until OD_600_ = 0.4–0.6 was reached. At this OD, they were cooled to 16°C and induced with 50 μM IPTG and incubated for 18–20 hours. Cultures were centrifuged at 10,000 rpm (JA10 rotor) for 10 minutes and the cell pellets were stored at -20°C until further use [6].

### Heterologous expression and protein purification of selected bacterial TSs

One litre of grown cells of *E*. *coli* BL21 (DE3) or ArcticExpress (DE3) harbouring a pET-TS plasmid (S3 Table) were defrosted and re-suspended in 10 ml buffer A (binding buffer: 25 mM Tris-HCl, 0.5 M NaCl, 20 mM imidazole, 5 mM MgCl_2_ and 1 mM Tris (2-chloroethyl) phosphate (TCEP), pH 7.8) and sonicated on ice using a 50% duty cycle at 50% power for 5 mins. Cell debris was removed by centrifugation at 40,000 g for 30 mins at 4°C to separate the soluble protein fraction from the insoluble fraction. His-tagged recombinant proteins were purified by Ni-NTA affinity chromatography (Qiagen). Bound fractions were washed with binding buffer (2 × 10 mL/L culture) and eluted with buffer B (10 mL/L culture; 25 mM Tris-HCl, 0.5 M NaCl, 0.5 M imidazole, 1 mM MgCl_2_, and 1 mM TCEP, pH 7.8) as described [6]. The obtained fractions were analysed by SDS-PAGE to confirm the purity. The pure fractions were pooled and desalted using a PD-10 column according to the manufacturer’s instructions [34].

### FPP and GPP synthesis

Farnesol or geraniol (150 mg) were dissolved in trichloroacetonitrile (0.6 mL) and stirred for 30 mins. Acetonitrile (20 mL) was then added followed by the ammonium phosphorylation salt (0.7 g) which was added in aliquots over 10 mins. The reaction was stirred for 4 hrs and ran a TLC using propanol/concentrated ammonia/water, 6/2/1 solvent system and stain with phosphomolybdic acid (PMA). The organic phase (ether layer) was washed with 1 M aqueous ammonia (3 x 30 mL). The ammonia washes were combined and washed 3 times with fresh diethyl ether (3 x 100 mL). The aqueous layer was reduced to a residue using a rotary evaporator and purified using silica gel. 50 mg of FPP and 50 mg of GPP was synthesized to use for the *in vitro* enzyme activity analysis.

### *In vitro* enzyme assays, compound extraction

All enzyme reactions were performed with freshly prepared protein. For mono-terpenoid (C_10_) and sesqui-terpenoid (C_15_) enzyme assays: 10 μg of purified TS protein was incubated with 100 μM of GPP or 75 μM of FPP in 1 mL of 25 mM Tris-HCl (pH-7.8) with 5 mM MgCl_2_ (33). A 20% (v/v) organic layer (nonane for mono- and sesqui-terpenoids, *n*-hexane for di- and sesqui-terpenoids) was added to the reaction mixtures to trap the volatile terpene products, followed by incubation at 28°C for overnight with continuously shaking at 50 rpm. After incubation, the organic layer was removed and the reaction mixture extracted twice with 1 mL of hexane, dried over anhydrous MgSO_4_ and further concentrated to 100 μL before analysis by gas chromatography—Quadrupole Time-of-Flight Mass Spectrometry (GC-QToF).

### *In vivo* terpenoid production and extraction

Due to GGPP insolubility in the preferred assay buffer, identification of TS activity with GGPP was performed in *in vivo* conditions. For *in vivo* diterpenoid production, cells containing pBbA2k-*EcispA*M22 plasmid with the selected TS (S3 Table) were grown in 2xYT with 0.4% (w/v) glucose until OD_600_ = 0.6 and induced with 50 nM anhydrous tetracycline (aTc) overlaid with an 20% (v/v) nonane, incubated at 30°C for 36 hours. For *in vivo* production of mono- and sesqui-terpenoids the cells containing pBbB2a-GPPS-TS and pMVA plasmid were grown in TB media with 0.4% glucose (w/v) and cells were induced with 50 μM of IPTG and 50 nM anhydrous tetracycline (aTc) at OD = 0.6 overlaid with 20% (v/v) nonane, incubated at 30°C for 48 hrs. Nonane layers were harvested and clarified by centrifugation (14,000 rpm, 3 min and 4°C), dried over anhydrous MgSO_4_ and analysed by GC-QTOF.

### Compound GC-MS analyses

The extracted terpenoids were subjected to GC–QToF (Agilent 7020) equipped with an Agilent Technologies 5977A MSD (Mass Selective Detector). The products were separated on HP5 capillary column (30 m, 0.25 mm i. d., 0.50 μm film, Agilent) using a temperature program of 50–280°C with a temperature gradient of 20°C/min and hold for 5 min at 280°C for sesquiterpenoids. For analysis of monoterpenoids, a temperature program of 50–230°C with a temperature gradient of 20°C/min and hold for 2 min at 230°C was used. For diterpenoids analysis a temperature program of 50–300°C with a temperature gradient of 20°C/min and hold for 5 min at 300°C was used. The injector temperature was set at 250°C with a split ratio of 10:1 (1 μL injection). The carrier gas was helium with a flow rate of 1 mL/min and a pressure of 5.1 psi. The ion source temperature of the MS was set to 250°C, and spectra were recorded from m/z 50 to m/z 450 with electron impact mode (70 eV). Compound identification was carried out using authentic standards and comparison to mass spectra to library spectra [35], and NIST (National Institute of Standards and Technology) library of MS spectra and fragmentation patterns, as described previously [36]. Authentic standards 3-carene (115576-25ML), limonene (183164-5ML), linalool (L2602-5G), ocimene isomers (CRM40748), nerolidol (H59605) and geosmin (G5908-1ML) purchased from Sigma-Aldrich were used to validate the compounds produced in *in vitro* and *in vivo* conditions.

### Ethical statement

As per our knowledge we have provided all the data and followed the ethical guidelines.

## Results and discussion

### Identification of TS homologues

TSs from different origins show substantial differences in overall primary amino acid sequence, but possess a strongly conserved metal binding domain consisting of an acidic amino acid (AA)-rich motif (D/N) DXX (D/E) or DDXXXE located within 80–120 or 230–270 AA of the N-terminus and an Asn/Ser/Glu triad closer to the C-terminus, which are the signature domains of the class I TS. 2,167 protein sequences out of 73,714 proteins were identified to be TSs and their homologues based on the HMM search as described in the Material and Methods. In a previous study, 262 presumptive bacterial TSs were identified and 27 proteins were functionally characterised in *in vivo* conditions [37]. Several other bacterial TSs have been characterised and their activities identified with single or multiple substrates [24,28,36,38–41]. In this study, presumptive TS homologue protein sequences were clustered by pairwise similarity, and from the resulting neighbour-joining tree (Fig 1) we identified TSs from Gram-positive bacteria, mainly from the order *Actinomycetales*, as well as Gram-negative bacteria belonging to numerous orders. We annotated 2,167 TSs in the neighbour-joining tree including geosmin synthases, 2-methylisoborneol synthases and several presumptive TSs whose functionality could not be assigned based on their protein sequences (Fig 1). TSs already identified in the MIBiG database (125 TSs; https://mibig.secondarymetabolites.org) [42] were marked when they had e-values of 0 by BLASTp search against the 2,167 HMM hit proteins. Many bacterial strains in the Proteobacteria and Firmicutes phylum have TS-like genes, while more than 70% contain the phytoene synthase motif. Enzymes containing this motif are mostly involved in phytoene biosynthesis, a tetra-terpene (C_40_) precursor for lycopene biosynthesis. Geosmin synthases and 2-methylisoborneol synthases from bacteria that have been well studied were excluded and presumptive TSs from Actinobacteria and proteobacteria were selected for functional characterization in this study. In total 22 TSs were selected from different clades (Fig 1) based on one of the following criteria: (i) the sequences contain a single terpene synthase domain (330–350 AA) which belongs to the Isoprenoid Biosynthesis C1 superfamily; (ii) they are from Gram-positive, Gram-negative, thermophilic or thermo-tolerant bacteria; (iii) they are from bacteria that contain other known TSs and have at least 30–40% identity to known TSs but its functionality are not characterized. Functional characterisation of these TSs and identification of their products based on their acyclic allylic diphosphate substrate specificity requires experimental validation which is shown in this work. The 22 potential TSs are selected from the orders of Actinomycetales (Actinobacteria), Burkholderiales (Proteobacteria), Myxococcales (Myxobacteria), Flavobacteriales (Flavobacteria) and Chloroflexales (Terrabacteria). They are: two TSs (AHY47823.1 and AHY45426.1) from the radiation resistant and thermotolerant *Rubrobacter radiotolerans* [43]; Four TSs (KYF56472.1, CAN96536, KYF59190.1, KYF75876.1) from a prolific secondary metabolite producer *Sorangium* sps [44]; TS KFG92939.1 from *Burkholderia paludis* which was isolated from Southeast Pahang tropical peat swamp forest soil in Malaysia [45]; AHH94051.1 from *Kutzneria albida* DSM 43870; KPH97530 from *Actinobacteria bacterium* OV450; ADU09376.1 from *Micromonospora* sp. L5; *geoS* (CAB41566.1) from *Streptomyces coelicolor* A3(2); ACY98649.1 from an aerobic, cellulolytic, thermophilic Gram-positive bacterium *Thermomonospora curvata* [46]; EXG82599.1 from *Cryptosporangium arvum* DSM 44712; ELQ82238.1 from *Streptomyces rimosus*; ABU58787.1 from *Roseiflexus castenholzii* DSM 13941; three hypothetical proteins (KFE96946.1, KFF23441.1 and EJL71407.1) from *Chryseobacterium* sps [47]; two (EST32145.1 and KIZ18902.1) from soil bacteria belonging to *Streptomyces* sps; CFM47198.1 from *C*. *vrystaatense*; KJK55818.1 from *Saccharothrix* sp. ST-888 (Table 1, S1 Fig).

### *In vitro* functional characterization of TSs

Codon optimized selected TSs were DNA synthesised and cloned into pETM11 with a TEV protease cleavable N-terminal His-tag by GeneArt (S3 Table). For the heterologous expression of these TSs, the plasmids were transformed into either *E*. *coli* Bl21 (DE3) or ArcticExpress (DE3) (S1 Table) and grown in 2xYT media with optimal inducer concentrations to obtain soluble protein. Recombinant proteins were purified using nickel affinity chromatography and subsequently salt and imidazole were removed by a desalting column prior to *in vitro* activity assays.

**Fig 2:**
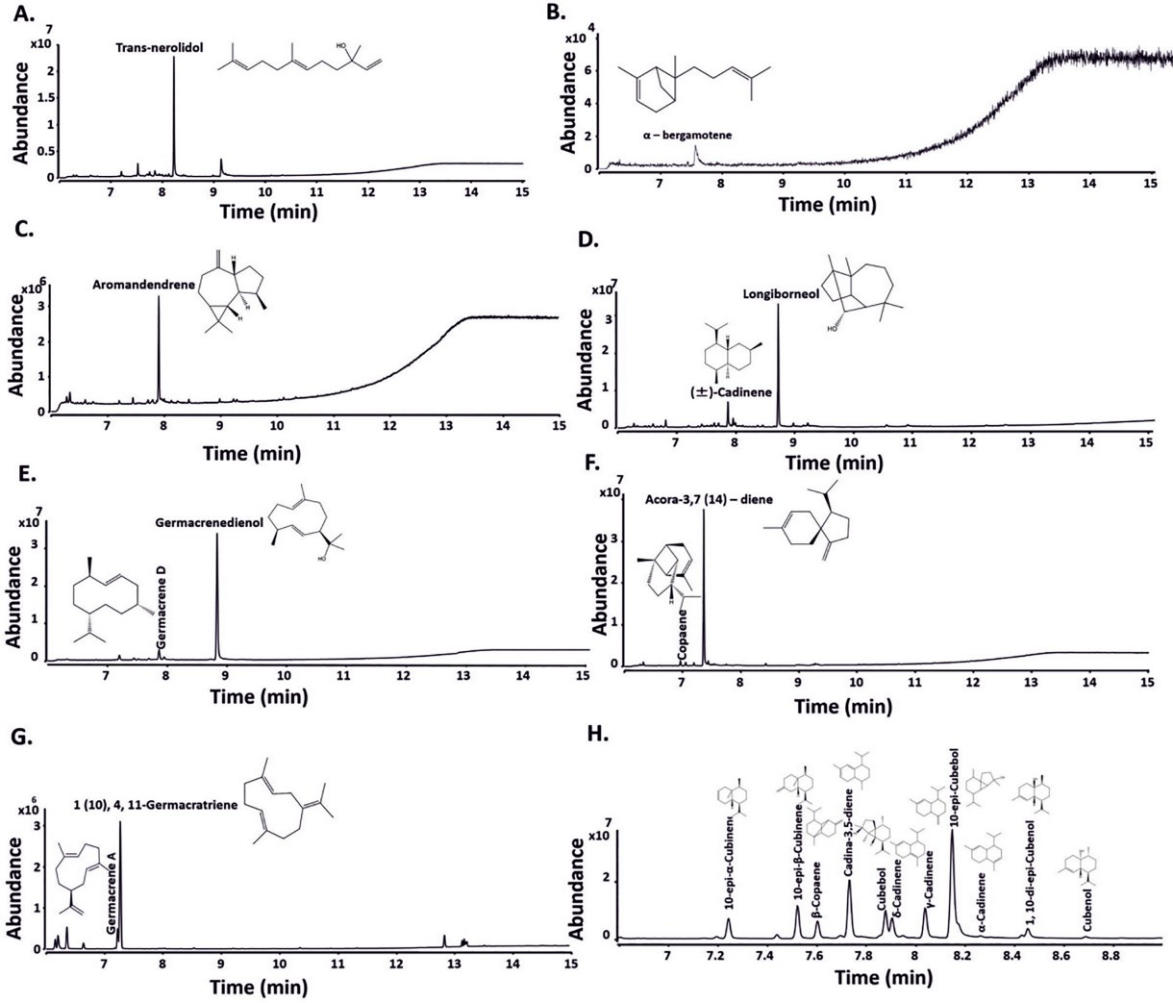
GC-QToF analysis of n-hexane extracts from *in vitro* assays obtained with selected TSs. GC-MS chromatograms of extracts profile with FPP are shown for **A.** AHY47823.1 (RrNerS) from *Rubrobacter radiotolerans*; **B.** AHY45426 (RrBerS) from *R*. *radiotolerans*; **C.** KYF56472.1 (ScAroS) from *Sorangium cellulosum*; **D**. KFG92939 (BpLonS) from *Burkholderia paludis*; **E.** AHH94051.1 (KaGerS) from *Kutzneria albida* DSM 43870; **F.** KPH97530.1 (AbAcoS) from *Actinobacteria bacterium* OV450; **G.**ADU09376.1 (MiGerS) from *Micromonospora sp*. L5.; **H.** CAN96536 (ScEpiS) from *Sorangium cellulosum*. The name and structure of the produced terpenoids are indicated in each trace.

**Fig 3:**
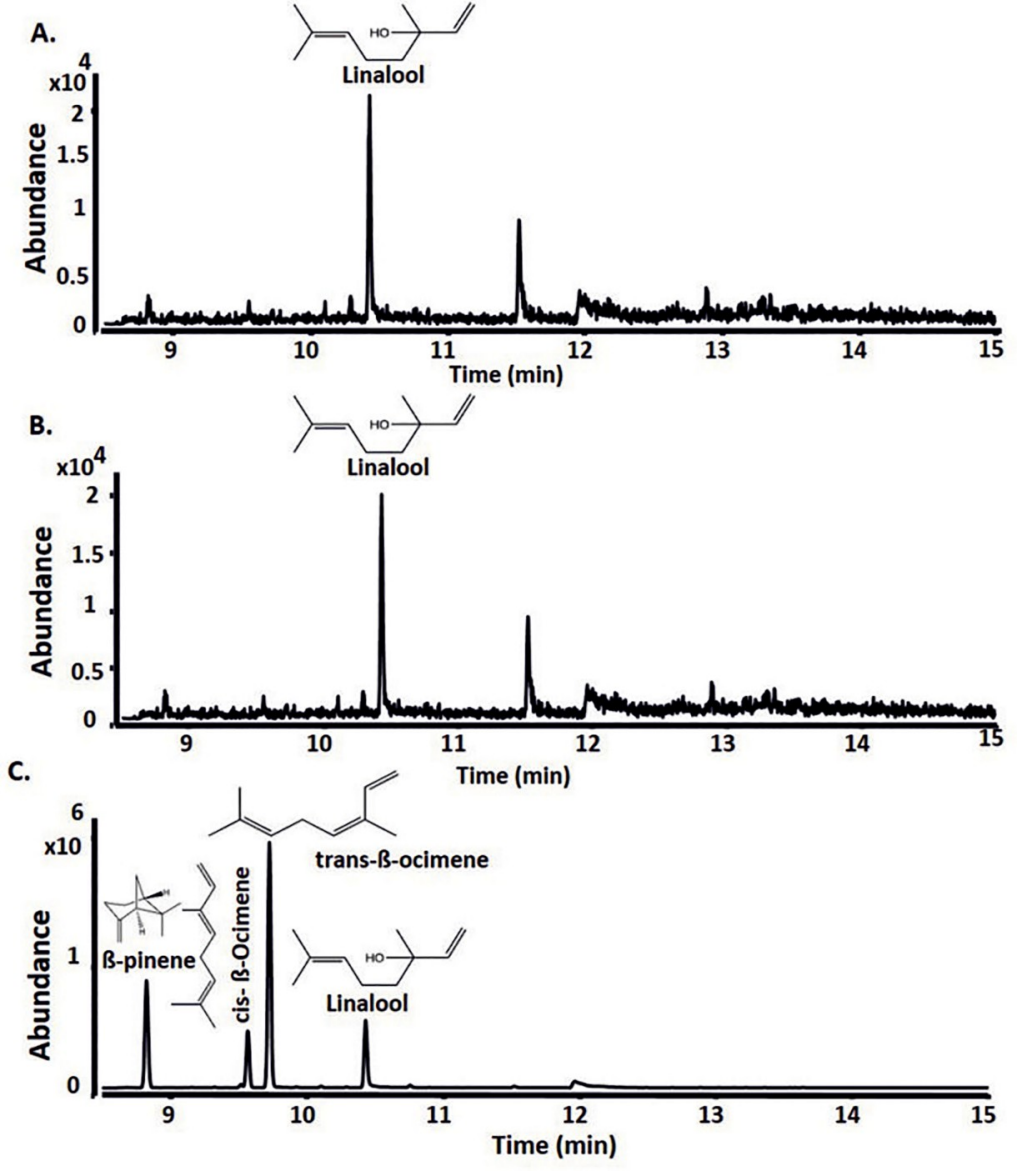
GC-QToF analysis of nonane extracts from *in vitro* assays obtained upon incubation with GPP for 16 hrs at 28°C. **A.** AHY47823.1 (RrNerS) from *Rubrobacter radiotolerans*; **B.** AHY45426.1 (RrBerS) from *R*. *radiotolerans* and **C.** 10-epi-cubebol synthase (ScEpiS) from *Sorangium cellulosum So ce56*. Other small peaks could not be annotated using the NIST library search.

Terpenoids resulting from the incubation of the purified recombinant TSs from different bacteria with FPP were extracted using hexane (Fig 2; Table 1), while products formed with GPP were extracted using nonane (Fig 3; Table 1). Products were identified using authentic standards where possible. Purified recombinant geosmin synthase from *Streptomyces coelicolor* A3(2) [48] and crude extracts with overexpressed limonene synthase from *Mentha spicata* (LimS) [29] were used as a positive control to validate the *in vitro* assays for sesquiterpenoids and monoterpenoids, respectively. As expected, purified geosmin synthase yielded the sesquiterpenoids geosmin, germacradienol and germacrene D in the assay mixture upon incubation with FPP (S2 Fig and S3 Fig), whereas the monoterpene limonene was formed when GPP was added to limonene synthase containing crude extracts (S4 Fig and S5 Fig).

Incubation of a recombinant TS (NCBI accession number AHY47823.1, RrNerS) from the radiation resistant, thermotolerant actinobacterium *Rubrobacter radiotolerans* [43] with FPP yielded a single product that was identified as trans-nerolidol by GC–QToF analysis (Fig 2A, Table 1) and this was further confirmed using standards (S6 Fig). Incubation with GPP yielded both R- and S- linalool isomers, which were confirmed using standards (Fig 3A and S7 Fig, Table 1). Therefore, this TS was annotated as linalool/nerolidol synthase.

Another terpene cyclase homologue, AHY45426 (RrBerS), from *R*. *radiotolerans* also showed activity with both FPP and GPP, yielding α-bergamotene (Fig 2B, S10A Fig, Table 1) and linalool (Fig 3B, S8 Fig, Table 1) respectively.

Therefore, these two TSs are the first to be identified in thermotolerant bacteria, *R*. *radiotolerans*, which have both mono- and sesqui-terpene synthases activities and are active up to 60°C (S9 Fig). These thermostable TSs can aid in engineering industrially important TSs to tolerate cultivation at higher temperatures [50].

The terpene cyclase (KYF56472.1, ScAroS) from the soil-dwelling Gram-negative bacterium *Sorangium cellulosum* converted FPP into only aromadendrene in *in vitro* analysis (Fig 2C and S10B Fig; Table 1). Aromadendrene is mostly observed in eucalyptus oil and is also produced by a citrus TS, *CsSesquiTPS5* [51] and it has been shown to have antibacterial activity to multidrug resistant Gram-negative bacteria [52]. KYF56472.1 was therefore named aromandendrene synthase.

The sesquiterpene cyclase (KFG92939, BpLonS) from *Burkholderia paludis* was incubated with FPP to yield two sesquiterpenoids, (±)-cadinene and longiborneol (Fig 2D and S10C Fig and S10D Fig; Table 1) and was therefore assigned as longiborneol synthase. Incubation with GPP did not yield any observable products. Longiborneol synthases are mostly found in fungi [53] and Norway spruce [54]. Fusarium uses longiborneol as a precursor for producing the tricyclic mycotoxin culmorin [55]. This enzyme from *B*. *paludis* is the first bacterial longiborneol synthase identified.

AHH94051.1 (KaGerS*)* from *Kutzneria albida* DSM 43870 has 50% identity to germacradienol/ geosmin synthase (fragment) and we tested its functionality with FPP in *in vitro* conditions. This yielded germacradienol and germacrene D (Fig 2E and S10E Fig; Table 1) and did not yield any monoterpenes when incubated with GPP. Production of germacradienol was confirmed by comparison to spectra published by Agger et al (2008) [56].

The hypothetical protein 450_5823 (KPH97530.1, AbAcoS) from *Actinobacteria bacterium* OV450 converted FPP into copaene and acora-3,7(14)-diene (Fig 2F and S10F Fig and S10G Fig; Table 1), while incubation with GPP did not yield any products. KPH97530.1 was annotated as acoradiene synthase based on its product formation.

TS (ADU09376.1, MiGerS) from *Micromonospora sp*. L5 incubated with FPP yielded β-elemene, and 8-isopropenyl-1,5-dimethyl-1,5-cyclodecadiene (or also called 1(10),4,11-germacratriene) (Fig 2G and S10H Fig and S10I Fig; Table 1). The sesquiterpene hydrocarbon, β-elemene, found is most likely an artefact of GC-MS analysis arising from thermal cope rearrangement of germacrene A at elevated temperature [57–59]. Therefore we assumed this synthase produced germacrene A in addition to germacratriene, and was annotated as germacatriene synthase, as germacatriene was the major compound produced.

Incubation of the sesquiterpene synthase (10-epi-cubebol synthase; CAN96536.1, ScEpiS) from *Sorangium cellulosum* Soce56 with GPP yielded the monoterpene products cis- and trans-β-ocimene, which differ in the position of the isolated double bond, and linalool which was not shown in previous work (Fig 3C) [49]. All three monoterpene products were confirmed using commercial standards (S11 Fig). When ScEpiS was incubated with FPP the products obtained were 10-epi-α-cubinene, 10-epi-β-cubinene, β-copaene, cadina-3,5-diene, cubebol, γ-cadinene, δ-cadinene, sesquiterpene alcohol 10-epi-cubebol, α-cadinene, 1,10-di-epi-cubenol and cubenol (Fig 2H). These results confirmed previous observations [49] The other minor compounds previously observed: germacrene D, (+)-eremophilene, bicyclogermacrene, T-cadinol, and α-cadinol were not observed due to the solvent extraction method used in this work, instead of sensitive closed loop stripping analysis (CLSA) method used in the previous study. As we have shown in this study, others have observed that heterologous expression of the epi-cubebol synthase from *Streptosporangium roseum* in *E*. *coli* also resulted in the production of β-pinene, β-ocimene (cis- and trans-) and linalool isomers, demonstrating that many sesquiterpene synthases can also accept GPP as substrate [60].

Until now, only two bacterial monoterpene synthases linalool synthase (bLinS) and 1,8-cineole synthases (bCinS) from *Streptomyces clavuligerus* are well studied [61] and structurally characterized [6]. Broad substrate spectra have been observed previously for bacterial TSs: spata-13, 17-diene synthase is an enzyme with sesqui-, di-, and sester-terpene synthase activity [41], a bLinS from *S*. *clavuligerus* accepts GPP and FPP [23], and incubation of corvol ether B synthase (BAJ27126) from *Kitasatospora setae* with GPP yielded linalool as a major product and small amounts of several acyclic and cyclic monoterpenes [62].

CFM47198.1, ELQ82238.1, ACY98649.1, EXG82599.1, ABU58787.1, EJL71407.1, KFE96946.1 were tested for compound production with FPP and GPP but no terpenoids were detected. (S12 Fig)

### *In vivo* production of mono- and sesqui-terpenoids by enhancing MVA pathway precursor supply

**Fig 4:**
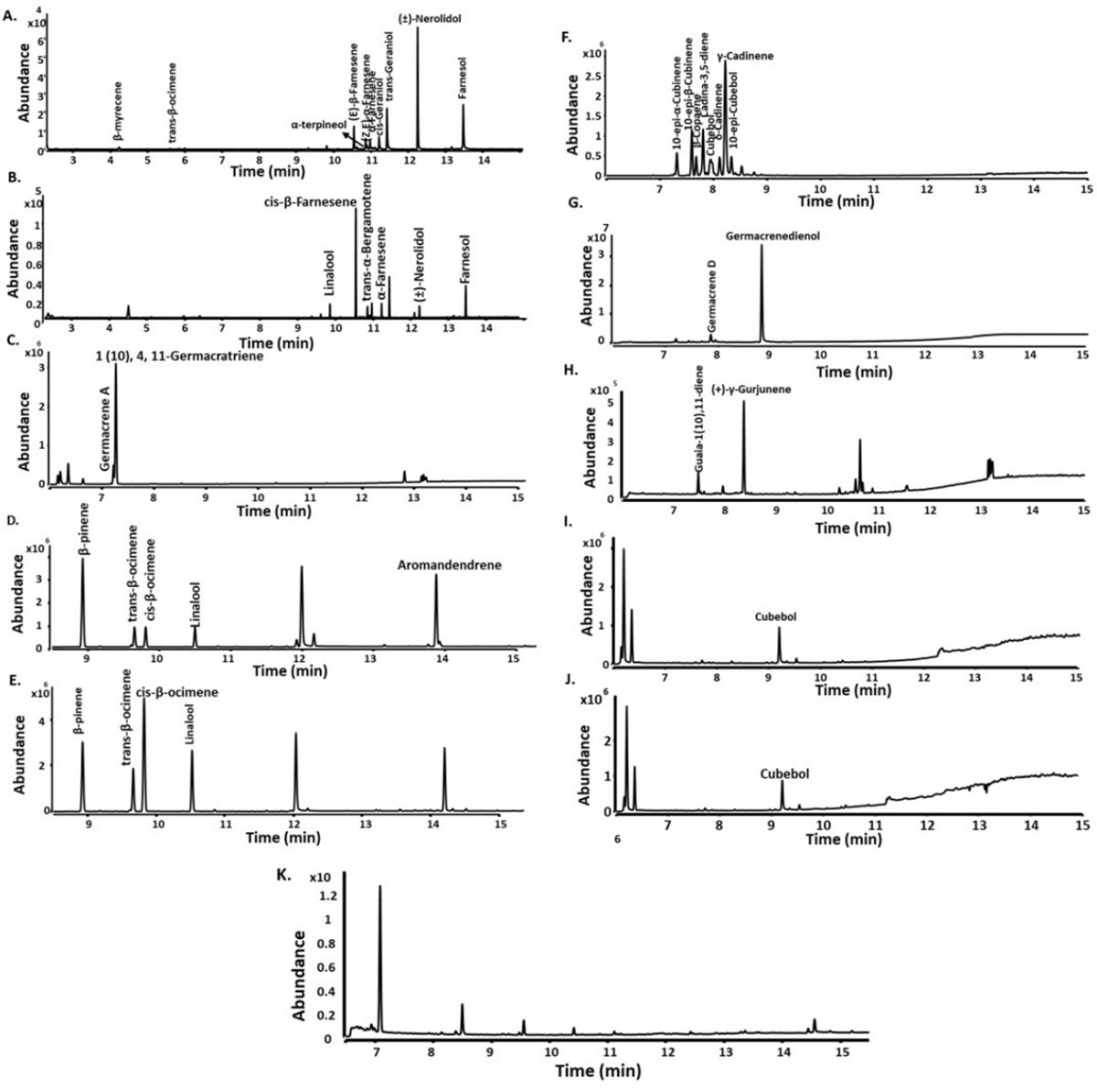
GC-QToF analysis of nonane extracts containing mono- and sesqui-terpenes by TSs produced in *E*. *coli*. **A.** AHY47823.1 (RrNerS) from *Rubrobacter radiotolerans*; **B.** AHY45426 (RrBerS) from *R*. *radiotolerans*; **C**. ADU09376.1 (MiGerS) from *Micromonospora sp*. L5.; **D.** KYF56472.1 (ScAroS) from *Sorangium cellulosum*; **E.** monoterpenoids from CAN96536 (ScEpiS) from *Sorangium* sps.; **F**. sesquiterpenoids from CAN96536 (ScEpiS*)* from *Sorangium* sps.; **G.** AHH94051.1 (KaGerS) from *Kutzneria albida* DSM 43870; **H.** ELQ82238.1 (SrGuaS) from *Streptomyces rimosus;* **I.** ACY98649.1 (TcCubS) from *Cryptosporangium arvum* DSM 44712; **J.** EXG82599.1 (CaCubS) from *Thermomonospora curvata DSM 43183;* and **K.** Negative control (pMVA+pBbB2a-GFP). Peaks were annotated using NIST library spectra.

To quantify the *in vivo* product formation, all identified TSs were cloned into the pBbB2a-GPPS (as described in material and methods) backbone plasmid and co-expressed with pMVA plasmid in *E*. *coli* [7] and FPP is supplied via endogenous IspA activity [63]. Cultures were overlaid with nonane to extract the metabolites and analysed by GC-QToF. RrNerS produced monoterpenes β-myrcene, linalool, cis-geraniol; and sesquiterpenes: (±)-trans-nerolidol, α/β-farnesene and farnesol in *in vivo* conditions (Fig 4A) whereas its homologue RrBerS yielded monoterpenes β-myrcene, linalool, trans-geraniol and trans-α-bergamotene, α/β-farnesene, (±)-trans-nerolidol, farnesol (Fig 4B). *MiGerS* from *Micromonospora* sp. L5 expressed in *E*. *coli* produced germacrene A and 1,(10),4,11-germacratriene as seen in the *in vitro* assays (Fig 4C).

ScAroS and ScEpiS both from *Sorangium*, produced similar mono-terpenoids, which are β-pinene, cis- and trans-ocimene, and linalool (Fig 4D and Fig 4E). However they produce different sesquiterpenes in both *in vitro* and *in vivo* conditions: aromandendrene is produced by ScAroS whereas 10-epi-α/β-cubinene, β-copaene, cadina-3,5-diene, cubebol, δ/γ-cadinene and 10-epi-cubebol are produced by CAN96536 (Fig 4D and Fig 4F). KaGerS from *Kutzneria albida* DSM 43870 produced germacrene D and germacrenedienol (Fig 4G).

Overexpression of hypothetical protein ELQ82238.1 (SrGuaS) from *Streptomyces rimosus* produced multiple sesquiterpenes: guaia-1(10)11-diene and (+)-γ-gurjunene when expressed in *E*. *coli* (Fig 4H, S14A Fig and S14B Fig). Whereas ACY98649.1 (TcCubS) from *Thermomonospora curvata* DSM 43183 (Fig 4I, S14C Fig) and EXG82599.1 (CaCubS) from *Cryptosporangium arvum* DSM 44712 (Fig 4J, S14D Fig) produced minor amounts of cubebol in *E*. *coli*. Terpene synthases may produce different terpene skeletons when expressed natively or in heterologous host compared to their product biosynthesis in *in vitro* conditions due to variations in protein folding and assay conditions [64].

Geosmin synthase from *Streptomyces coelicolor* A3(2) when expressed in *E*. *coli* produced various monoterpenes: β-myrcene, β-ocimene, linalool and geraniol (S13 Fig) which was unexpected and unexplored for this enzyme as well as the sesquiterpenes: germacradienol, germacrene D but geosmin was not detected, presumably due to inactivity of the C- terminal a-domain in *in vivo* (S13 Fig).

### *In vivo* production of possible diterpenes

**Fig 5:**
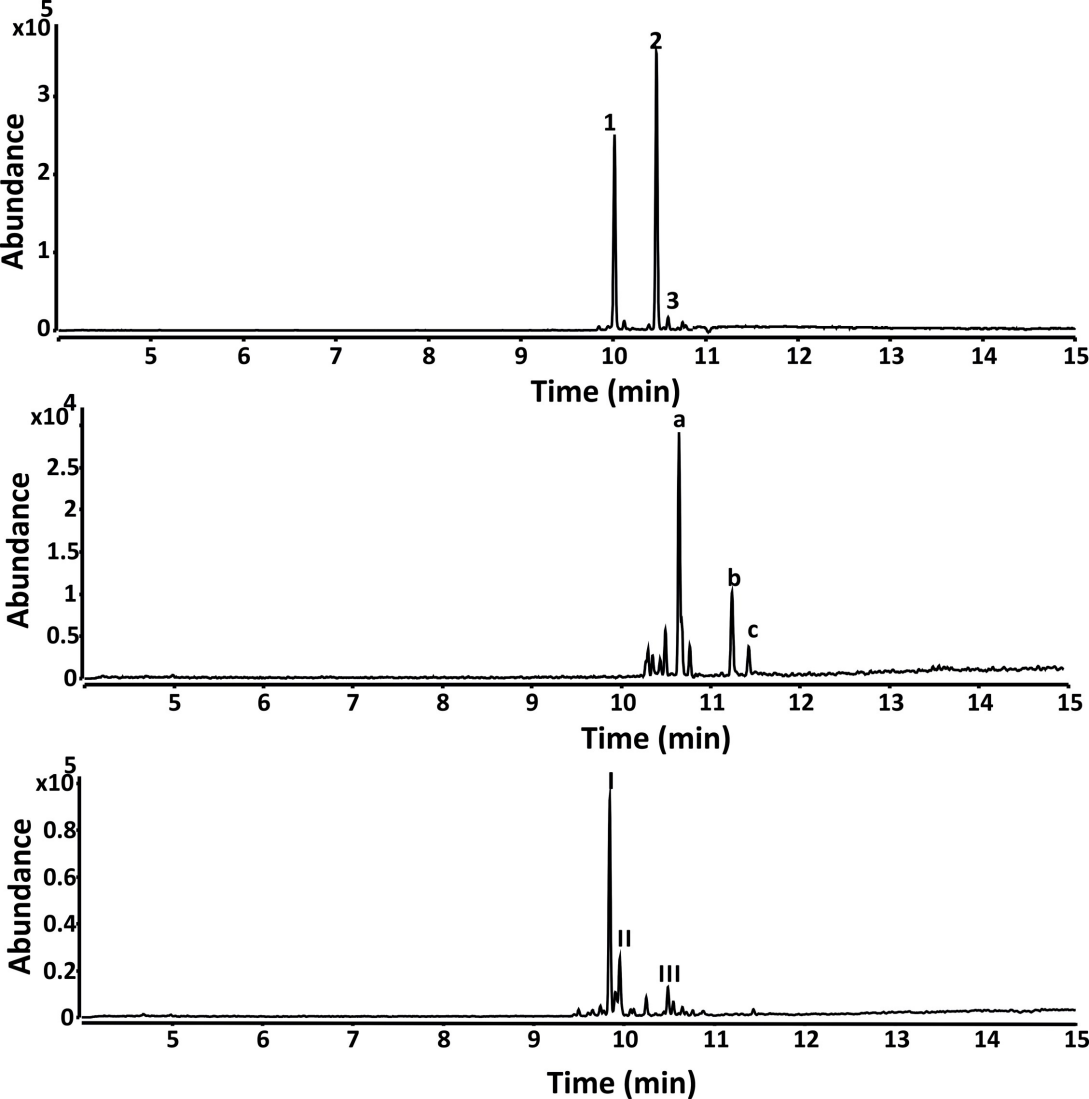
GC-QToF analysis of nonane extracts containing possible diterpenes from *E*. *coli in vivo* production expressing the following TSs. **A**. ABU58787.1 from *Roseiflexus castenholzii* DSM 13941 (RcDTPS), **B.** EJL71407.1 from *Chryseobacterium sp*. *CF314* (CsDTPS), and **C.** KFE96946 from *Chryseobacterium luteum* (ClDTPS). Numbered peaks in each Fig correspond to compounds produced. The mass spectrum for each compound is in S16–S18 Figs.

For diterpene synthase activity, the substrate GGPP was synthesized in house but due to its hydrophobic nature the substrate was insoluble in aqueous solution and could not be used in *in vitro* enzyme assays. For rapid identification of diterpene synthase activity it is essential to generate abundant amounts of GGPP for *in vivo* production in *E*. *coli*. For this purpose, the native farnesyl pyrophosphate synthase (*ispA)* from *E*. *coli* with two mutations D2G, C155G (*ispAM22*), which can function as geranylgeranyl diphosphate synthase [65], was employed to generate GGPP *in vivo*. GGPP synthase activity using the variant prenyltransferase was confirmed by overexpression of spata-13, 17-diene synthase from *Streptomyces xinghaiensis* [41] together with GGPP synthase (*ispA*M22). This yielded the diterpene spata-13,17-diene, where the produced compound was extracted using a nonane layer (S15 Fig). ABU58787.1 (RcDTPS*)* from *Roseiflexus castenholzii* DSM 13941 (Fig 5A, S16 Fig), EJL71407.1 (CsDTPS) from *Chryseobacterium sp*. CF314 (Fig 5B, S17 Fig, and KFE96946 (ClDTPS) from *Chryseobacterium luteum* (Fig 5C and S18 Fig) produced various possible diterpene products when expressed together with *ispA*M22 in *E*. *coli* DH5α. Due to low product yields, the detected compounds could not be annotated by a NIST library search alone. By co-expressing the heterologous MVA pathway, the precursor supply for diterpene, production was enhanced which led to a 10-fold increased production. However, this was still not enough to determine the compounds produced. Large scale production and purification of the diterpenes would be required for structural determination by NMR analysis.

In this study, we identified 15 putative bacterial TSs out of 22 that were tested, which produced structurally diverse mono-, sesqui- and di-terpenoids by *in vitro* and/or *in vivo* assays. We have shown TSs with new activity: 7 sesqui-terpenoid synthases, 3 di-terpenoid synthases and 5 mono-/sesqui-terpenoid synthases which were identified from Gram-positive, Gram-negative and thermophilic tolerant bacterial species. Many sesquiterpene synthases were shown to also have activity as a monoterpene synthase, which suggests that dual substrate specificity is very common for bacterial TSs.

*In vivo* production of all types of terpenoids by *E*. *coli* makes an attractive platform for rapid identification of enzymes as well as for better and cheaper production yields through metabolic engineering. Especially the newly discovered TSs from the thermophilic/tolerant bacterial species is very promising for further protein evolution studies to design the end terpene product, which we have started [50] and to exploit the enzyme stability at higher temperatures. In addition, production of diterpenoids in *E*. *coli* as an alternative screening method to *in vitro* assays is useful due to the difficulties with solubility of GGPP.

The results presented in this study suggest that further exploration of putative TSs from different bacteria along with Actinomycetes could expand the structural plethora of terpenes. The majority of the terpene products identified in this study are known to be produced by plant or fungi and reveals that TSs are widely distributed in bacteria. Given the number of uncharacterized bacterial enzymes that exist in nature, it is likely that there remains a wealth of chemistry to be discovered and exploited. Expanding the search for novel terpenoid biosynthesis will provide numerous structures with unexplored properties that could potentially help to develop novel compounds for pharmacological or industrial applications.

## Supporting information

S1 Table Strains and primers used in this study. *E*. *coli*, DH5a cells were used for plasmid propagation and Bl21 (DE3) and Arctic Express (DE3) cells for recombinant protein expression.
(DOCX)

S2 Table Protein sequences of selected terpene synthases. (DOCX)

S3 Table Plasmids used in this study. (DOCX)

S4 Table List of terpene synthases annotated in phylogenetic tree, Fig 1. Known TSs annotated in the tree in Fig 1 where the branches were indicated in colour based on their functionality: monoterpenoids (light blue), 2- methyisoborneol (orange), sesquiterpenoids (purple) and diterpenoids (blue). The same colour scheme is adapted here.
(DOCX)

S1 Fig Bacterial terpene synthases neighbour-joining tree. Neighbour-joining tree constructed from the amino acid sequences of characterized terpene synthases in this study (underlined) and in previous studies. Geosmin synthases (dark red), 2-methylisoborneol synthases (orange), monoterpene (light blue), diterpene (dark blue) and sesquiterpene synthases (purple) are shown and uncharacterized bacterial terpene synthases in this study (in red).
(DOCX)

S2 Fig GC-MS traces of geosmin standard. GC-MS traces showing the separation of geosmin (0.1 mg mL^-1^) on a HP5 column and its retention time at 7.5 minutes. B. Mass spectra of geosmin.
(DOCX)

S3 Fig GC-QToF analysis of n-hexane extracts obtained from *in vitro* assays. GC-MS chromatogram of geosmin synthase with 75 μM of FPP. Peak 1: geosmin (rt: 7.5), peak 2: Germacrene D (rt: 7.86), peak 3: germacradienol (rt: 8.84). B. Mass spectra of compounds observed in the extracts.
(DOCX)

S4 Fig GC-QToF analysis of limonene standard. A. GC-MS traces showing the separation of limonene (0.1 mg mL^-1^) produced in this study on a HP5 column. The internal standard, sec-butyl benzene (0.1%, v/v), has a retention time of 9.5 minutes. Peak 1: R- Limonene (rt: 9.41). B: Mass spectra of limonene.
(DOCX)

S5 Fig GC-QToF analysis of nonane extracts obtained from limonene synthase *in vitro* assays. **A.** GC chromatogram of 10 μg of limonene synthase (pJBEI-6410) crude extracts with 75 μM of GPP. Peak 1: limonene (rt: 9.41). B. Mass spectra of limonene.
(DOCX)

S6 Fig GC-QToF analysis of nerolidol standard mix and products obtained by AHY47823 upon incubation with FPP. **A.** GC-MS traces showing the separation of standard nerolidol mix (0.1 mg mL^-1^) on a HP5 column. **B.** MS spectra of -cis and -trans Nerolidol. **C**. GC-MS chromatogram of trans-nerolidol produced by AHY47823 with FPP. **D.** Mass spectra for trans-nerolidol produced by AHY47823.
(DOCX)

S7 Fig GC-QToF analysis of linalool standard mix and products obtained by AHY47823 upon incubation with GPP. A. GC-MS traces showing the separation of standard linalool mix (0.1 mg mL^-1^) on a CP-Chirasil-DEX-CB column. **B.** GC-MS chromatogram of linalool isomers produced by AHY47823 with FPP. Samples were analyzed by GC on an Agilent Technologies 7890A GC system equipped with an FID detector, a 7693 auto sampler, and a CP-Chirasil-DEX-CB column (25 m × 0.25 mm i.d., 0.25 μm film thickness). For linalool, the program initiated at a temperature of 70°C which was then increased to 90°C at 8°C/min. This was followed by an increase in temperature to 150°C at a rate of 2°C/min and then to 190°C at 40°C/min (1 min hold). The FID detector was maintained at a temperature of 200°C with a flow of hydrogen at 30 mL/min.
(DOCX)

S8 Fig GC-QToF analysis of linalool standard mix and products obtained by AHY45426 upon incubation with GPP. A. GC-MS traces showing the separation of standard linalool mix (0.1 mg mL^-1^) on a CP-Chirasil-DEX-CB column. **B.** GC-MS chromatogram of linalool isomers produced by AHY45426 with FPP. Samples were analyzed by gas chromatography on an Agilent Technologies 7890A GC system equipped with an FID detector, a 7693 auto sampler, and a CP-Chirasil-DEX-CB column (25 m × 0.25 mm i.d., 0.25 μm film thickness). For linalool, the program initiated at a temperature of 70°C which was then increased to 90°C at 8°C/min. This was followed by an increase in temperature to 150°C at a rate of 2°C/min and then to 190°C at 40°C/min (1 min hold). The FID detector was maintained at a temperature of 200°C with a flow of hydrogen at 30 mL/min.
(DOCX)

S9 Fig Total ion chromatograms of products obtained from an incubation RrNerS (left) and RrBerS (right) with FPP for 3 hours at variable temperatures. Samples were analyzed by GC-QToF on HP5 column.
(DOCX)

S10 Fig Comparison of obtained mass spectra with NIST Library spectra. The reference spectra from the NIST library shown in blue and the compound spectra shown in red.**A.** α–bergamotene produced by RrBerS, **B.** Aromandendrene produced by ScAroS, **C and D**. cadinene and longiborneol produced by BpLonS, **E.** germacrene D produced byKaGerS, **F** and **G.** copaene and acora-3,7(14)–diene produced byAbAcoS, and **H** and **I.** β-elemene and 1(10),4,7(11)-germacra-triene produced by MiGerS.
(DOCX)

S11 Fig GC-MS traces of authentic monoterpenoid standards. **A.** GC-MS traces showing the separation of monoterpenoids (0.1 mg mL^-1^) standards used in this study on a HP5 column. The internal standard used, sec-butyl benzene (St; 0.1% v/v), has a retention time of 9 minutes. Peak 1: cis-ocimene (rt: 9.56), peak 2: trans-ocimene (rt: 9.72), 3: linalool (rt: 10.43). **B**. Comparison of obtained mass spectra with NIST Library spectra and standard as shown in A. of products yielded by CAN96536 with GPP in *in vitro* assay.
(DOCX)

S12 Fig GC-QToF analysis of n-hexane extracts from *in vitro* assays obtained with selected TSs. GC-MS chromatograms of extracts profile with FPP are shown for **A.** BpTPS; **B.** ScTPS1; **C.** CvTPS; **D.** SsTPS; **E.** ScTPS2; **F.** SniTPS; G.SnaTPS. The peaks in the chromatogram do not correspond to any terpenoids.
(DOCX)

S13 Fig Comparison of obtained mass spectra with NIST Library spectra of products yielded by geosmin synthase in *in viv*o conditions. **A.** GC-MS chromatogram for geosmin synthase products, **B.** mass spectra for Germacradienol, **C—G**. Comparison of obtained mass spectra with NIST Library spectra of products yielded by GeoS.
(DOCX)

S14 Fig Comparison of obtained mass spectra with NIST Library spectra of products yielded by TSs in *in vivo* conditions. **A–B.** SrGuaS **C.** TcCubS **D.** CaCubS.
(DOCX)

S15 Fig GC-QToF analysis of spata-13, 17-diene from nonane extracts of *in vivo* production in *E*. *coli*. **A.** spata-13,17-diene produced by overexpression of ispAM22 and WP_095757924 in *E*. *coli*. B. Mass spectra of produced spata-13,17-diene.
(DOCX)

S16 Fig Mass-spectra of diterpenoid compounds produced by RcDTPS from *Roseiflexus castenholzii* DSM 13941 corresponding to the peaks annotated in Fig 5A. (DOCX)

S17 Fig Mass-spectra of diterpenoid compounds produced by CsDTPS from *Chryseobacterium sp*. CF314 corresponding to the peaks annotated in Fig 5B. (DOCX)

S18 Fig Mass-spectra of compounds diterpenoid produced by ClDTPS corresponding to the peaks annotated in Fig 5C. (DOCX)
