# Supplementary material for: Exploring novel bacterial terpene synthases
Source: PLoS One. 2020 Apr 30;15(4):e0232220. doi: 10.1371/journal.pone.0232220 (PMC7192455; doi:10.1371/journal.pone.0232220)
Supplement: S1 Table — E. coli, DH5a cells were used for plasmid propagation and Bl21 (DE3) and Arctic Express (DE3) cells for recombinant protein expression. (DOCX) [file pone.0232220.s001.docx]

| **Strains** | | |
| --- | --- | --- |
| **Strain name** | **Strain Phenotype** | **Reference(s)** |
| DH5a | *E. coli* K-12, fhuA2 (argF-lacZ) U169 phoA glnV44 80 (lacZ) M15 gyrA96 recA1 relA1 endA1 thi-1 hsdR17 | New England Biolabs |
| BL21 (DE3) | *E. coli* B, fhuA2 [lon] ompT gal (λ DE3) [dcm] ∆hsdS λ DE3=λsBamHIo∆EcoRI-B int::(lacI::PlacUV5::T7 gene1) i21 ∆nin5 | New England Biolabs |
| Arctic Express (DE3) | *E. coli* B F^–^ ompT hsdS (rB^–^ mB^–^) dcm^+^ Tetr gal λ(DE3) endA Hte [cpn10 cpn60 Gent^r^ ] | Agilent Technologies |
| **Primer name** | **Sequence (5’-3’)** |  |
| pETM11-fw | GCCCTGAAAATAAAGATTC |  |
| pETM11-rev | TGAGATCCGGCTGCTAAC |  |
| pBbB2a-rev | caaagggatcctaaggatctccagg |  |
| GPPS-rev | CCCAAGCTTAATTCTGACGAAATGC |  |
| ispA_D2G-fw | tgatagagaaaagaattcaaaagatAGGAGGATAAAGAAATGAAACATC CCATCACCATCAcc |  |
| ispA_C155G-fw | ctaatgcctgaccaccgcccattc |  |
| ispA-rev | AGCAGCCGGATCTCAttatttattacgctggatgatgtag |  |
| TS_fw | gttGCATTTCGTCAGAATTAAAGGAGGATAAAGAAATGAAACATCACCATCACCATCAcc |  |
| TS-rev | gatccttaggatccctttgGGTGGTGGTGGTGCTCGAGTTA |  |

**S1 Table:** Strains and primers used in this study. *E. coli*, DH5a cells were used for plasmid propagation and Bl21 (DE3) and Arctic Express (DE3) cells for recombinant protein expression.
