# Supplementary material for: Exploring novel bacterial terpene synthases
Source: PLoS One. 2020 Apr 30;15(4):e0232220. doi: 10.1371/journal.pone.0232220 (PMC7192455; doi:10.1371/journal.pone.0232220)
Supplement: S2 Table — (DOCX) [file pone.0232220.s002.docx]

**S2 Table:** Protein sequences of selected terpene synthases

>AHY47823.1*_Rubrobacter_radiotolerans* MRGTAWPGLSYPFPPLINEHSDAVHAETVEWARSFGLMEEAGMREKVLATNIGRLAGRFHPSAPRERLRL

VSDWYAWMFFRDDLCDEAELGRRPDLLAAADLRYLGVLRGEEPGEEDGPLARAMRDLRERLLPVVPAALW

MRRFVRSVKEHFDSTLWEASNRVRREVPDLQTYTRMRPITGGMYVDADFIEITTGLYLPTEVRTDPTVSD

LTRMSNNAVCWANDIISLTKELASGDVHNIVLVLMAERDLSLEEAGRTAVGMHDREVTRFLALERELPSF

GRTIDENLARYVSVLRYRMRGNLDWSLESLRYRTV

>KYF56472.1*_Sorangium_cellulosum*

MPSERHSVVVSKGEVETFDYPFPASRNANVDIAERRTLQWIRRLQLAPEGRALSRLKATGFAQLAAWLLP

WANMRTLELASDFTAALFLLDDAYDEGDLSMDPEAVERLNEKYLGELFGYVEPDMSDPLTRGLLDVRDRI

KSSHPHFFLNRWLAHFQFYYEANLWEANNRRRARTPCVDEYLLMRRYSGAVYTYCDLLELLLERPLPLEV

VQHPTIQCVRDICNDILCWTNDYFSLGKELRSGDVHNLILVLRDNHAITLEEAIARLKQMHDERIAEYQD

VKEKVLALWDDEATRLYIGAADAMIAGNQRWALEARRYSGLESLIARAG

>KFG92939.1_*Burkholderia_paludis*

MKTDALKIELAALRIPVFDVPWAGACSPHAQRIETRMLEWADDHGLLVNDMYRKRVMRTRYGWLAARCYP

NADPALLQVIADYFVWYFLTDDLFIDRVETLGPGTLTHLVAIVDVLDYDQTARQPVYGERAWLDVCRRLR

ARLSAEHFARFAQGMRLWATTAGLQILNHIHAESVGIPQYETIRRHTSGMNPCLALADTANCGAVPPDTF

HRPDVQELCRHANHIVCWSNDIQSLGIEARQPGQFRNMVLIRRLEGHTLQEGVDYTAARVRDEIGEFVRC

ADALSQHADTRVRGLVDGCRYWIRGYLDWVARDTQRYAAAYADDADDRGLIAPSGSVARD

>AHY45426.1*_Rubrobacter_radiotolerans* MDPVRGGAGGVAVPPVVCRFDEGLSPHVGEVRKLHARWCERHGIYPSAAVGELFTAMDIGDLACRMHPGG

NAGDLALLADWSAWLLLRDDRWDAGEYLVEWERLAERDRVYLRLMRSGRTGEEPGSRRDGTRQDGLYTGL

EDLCARLRRRAADNGLPDPVSAGFLTTMGTFFRGSVRQAFLQRRREVPDLKEYLELRRVTGGLDILTHVR

AATDGFSLAGDALSGADARRLTLAADNICCWHNDLVSLNKELAGGEVNNLALVLVGDPASPCQTVEEGVE

AVVGMIYSEQDEFARLGELLRRRGGPHARTTAWYVKMLEERISGIISWQDRCSRYQVERLQGERYGSP

>KPH97530.1*_Actinobacteria_bacterium OV450*

MDTALSGEYVINGCSPADFAQAVGDIAPWMRQRLAEIYRPMASALADELNLWPIPGGIPGEYTFPPLPEH

IAKIDAEVQSWIDHHGLNPPGRTYPHPRLAHHVYAILHDAPVEVLRVQILWWERIIRHDDSTVEPGLLTA

SFPSEVDQILRNRVLPGEPDRHHLAFLDLRDAIADIDGGPTLLPTMADEAVALLQGSLTEQHYRTTGTVP

TLRQYLHYAADLIGIKDTIALGRLAPGGLLPGEIPPAFLPPLWRLATDIIRLTNDVIGAHRESVEGSPTV

FAAMAAQYDLAPADAARAVLALIDVLMRDFQHTIDEILPECPPKIHRELVLLQRRVCAAYAWDLTVISTR

YDPATRRREAR

>ADU09376.1_*Micromonospora sp. L5*

MRDFALSALHEPPFPSRRHPAVDQTAAESAAWVRDFGLVEDQAGRRRLAGAHAAELAARACPEASPSGLR

LLTDLVNWLFVVDDACDDDGLGATPTLLAPTLAALLAVLDRHGDPEAPAPADGGPIALALHDLCRRARDR

GRPAHLLRLVSQLREYLLALLWEAANRERRRVPGVAEYTQMRRHTGGVRPSFTVTDLARPSAPRADQRVA

PALTVLDALATDLICWCNDLFSYGKERGATPEAHNLVTTIAGETGQDESAALYAAADRFNKTLATYAERD

AALSAVADKGMRAFLDTRRDWIRATYDWSRAASRYA

>CAN96536.1*_Sorangium_cellulosum*

MHRALLCPFPATTPHPQAAQLANDCLEWTRKCGLLPDESPRTLDKVRSYSALAAHCYPDAHFERLRAICD

YYSWLFFFDDVCENTSLNGAEPKVVSSLLFDVYGVLRGPTAAVGHAPFAQALADIWRRIGDGCPGFWRRR

LIRHVENYIDGCVWEAQNRQLDRVPSRAVFEGMRMHTSTMYEFWDFIEYAGDLFLPDEVVEHPLVAEVRR

AGNAIASFANDIYSLRKETSNRDVHNLVVVLMHEERIELEAAYARAAGIHDAQVEHFLDLVKHLPTFSAT

IDRNLARYVEGIRIWIRANHDWSIVTPRYNEPDAR

>ABU58787.1_*Roseiflexus_castenholzii DSM 13941*

MMTIEPWYWQAVESINYPFPQRMHQAVSWLEDEIVTWCYVHKLIRDHAQEQHLRSALLAEFIARANAEIP

PSMLRLIGLWTVWFFLLDDLTDTLPSVEELADLQMRILTVVSEGRAYDYDHPLILAAADLSDLLKQCAGS

VTLVRFLRALAQTLEAHLWEVSTRLADIQPDSQTYTNMRLWSGAFFPMIALADMAQGSVLPSHIFEHAIV

QELIRAATQAVLWYNDLWSYPKEIQKHKMPHNIVHILAHENRIPLSEALSLTIAQHDRAIQQFLRLKKQI

HTLNGAEDVVLRFVESIEAWLRATQDWSHRTDRYRQQMVRAIHP

>KFF23441.1_*Chryseobacterium_vrystaatense*

MNTLELLNKQFNYPFPTLKNPNADQLQEITENQWIDGEYLWLYEQNPDLRKKYKKTKTAHIAAQWFPTAS

PERFKPICRLMLWTLYNDDLYEESVSGDIKNIHAQSIAVLNGEISAAESTIPLASMLASLRQELLEFIPQ

ESIARFTKMISRYFTGLETELIYKEKKMFPTIAECIALRENSICLYPFLQLTEVETGIVLPPEIHEHPVI

CRLQTLACHLVTYFNEVQSVVKDEATDSVYYNIVKVIQHERQISLEDACLKDLRLHNEDLKEFVALQASL

PDFGIWQDAVVNWVHYMSMVLSGWKNISTKLGRYNAMDFPEAKELKEKLNQM

>EJL71407.1_*Chryseobacterium sp. CF314*

MNVPLLESQLPIMIHPQMNAIVDDIKEYYKTDIFNISSENARLYGDHMSTGALYSSYIHPLGNIDKVKAV

SRYYCYWALMDDQFFDNSVDLDSIIQTFDGFQSALNEAPDIEKIFLPISEFCSRTDWTMETKEMFKSEMN

RYLESVLKLRTIEVQMKVVSLEEYLSYRAFDVAMNVIYSLAWYIQHDMPSSLYYSAEFEKIFEYSGISIG

LLLDLYTLKAKKKEIRNYAHAIRIIQRAENCDEEEAINKGVRLFYEYDSKLEEEFNRLEAKYPDAIRYFR

YIQSGSVKYCNESRKIRYKQVDDIDENLVKGRTVI

>ACY98649.1_*Thermomonospora_curvata* DSM 43183

MDAALVLSLADRVASLADRSGMHPAAQLIGAQAEGWARSRGLLLGDPDATPLGRARFERLACRIFPHAQP

DRVVLFARWLMWLFALDDHFDDTPLGASATSVDGLYADLLGALRRGHTKPEAGALELALEELWRDTVPGT

SPQWRHHFLRLMEEHRAACAEEAVNRRTGRIAPLADYPVLRRRSAGPFLYELAEPVLQVALDPRLKRSPA

WKALVDGTADMITWANDVVSYPKESRQGTVPVTGNLVAVACRELGMAPAQAASWVVDRIARRAPQVREAA

RAVGAELDRLEIGPQGRKDTAAVVRVLLQAPRAHMDWLAETGRYTPPVRSPVVLLHRTAAGVARTIG

>KJK55818.1_ *Saccharothrix sp. ST-888*

MNLPAPGPVLAPANRPRTGQTPRTPEEFAAAVVRHTGWVVARGLLPAQDVPRYQVFALHELIGHSYPRAR

GAELDLLLDILGWFTLLDDRFDGPTGYRPAEAHALIDPLLAVLRSPRPDVPAPDFPTSGLPAAAPDAAPD

AALIAAWQDLWHRQAGPMTDTWRRRAAADWRACLTTFVTETLHRARGTLPGLSETALLRRHASCLYPFMN

MLERVHGTGAAAQLHAEPSLHRLRAQTADAATLINDLFSLEREERQSAAQFNMVMTLQRTCGYRRDEAIT

AVRTKVRRIQDDSDALRRQLVRRHPAGHWYLHGTRELVDGVYTWTSTSHRYHGH

>KYF75876.1_*Sorangium_cellulosum* MGSELAAQRYLSNDIAGLMARWIPGAVGPDLDVTIDGVIVATLLDDQIDSGLADQPEEVARICQAMLSVI

RPDAAAPRSPSKPLINAFADVWGRLTQGRTATFCARTARHWQWYLDAYVDEAKNRNRAIMPTRAEHFELR

RRSGFVYAMMDMTERAYQFEAPELAWHTPQIQSMLTITADVVDTLNDLHSLEKEEGRGDLHNFVLVVQHE

RRCSRAEAIAETRDSVHEWIASFIKLTEEIPELCASLGLTEPERDNVRQLVTGMKTAMRGYFDWSCKTDR

YATTNLVRAGQAAYPHDLL

>KYF59190.1*_Sorangium_cellulosum*

MNPRSGFGASQGSGGAEGFGERLKGMELNFSGFEPVRTPALLVPVEHRVSEHLEQIRSPYVDWVVDAKLV

EPGTRGYDVLMRTKLDDCAALIFPDHPRELVLYGAISLALFFALDDVVDDVNADRSRKLAYIRRVEQIAS

GDAPAARDDHIVRAWHRWFEEIEALASPRAFEGFAGALRFHLRALRAQALNQQSAAPCPTTHLMRRRDNV

GSSYFMPLAAIFLEREHGLSMQEVLEDQHVKGITDLAAFVIVLHNELLGLYKDMRSGEANFVELLRREHG

IELQSACDLAGKLADDMVKAMVQMATDLSALVDGYEEKAEAIARCVRTAYSLIRGTFDWYMITRRYYDEE

CFSM

>EXG82599.1*_Cryptosporangium_arvum_DSM_44712* MTATTSPNPAATTGSARTAVRVVRLLRALRRWAAEVGPSFADAVEIDEVSAMVLVDAGPHAPTGELLAPA

MTAVWMQVADDHVDRFTHDQAEFDGLIQRWRSVSAGARPTPGDPLERGLADLVRRLRTAPRYPSLFPVWR

RSFGRMLDAWVFEQTTATRLARGEPAPSIESYLAHHHSFGLRPIVLAWWVTAGGDDLPDALEALLAALDE

IETAVRLANDLVMADRARTEPGSVDALLLGADATWVSDRATEHLRRGHDLLAPLIETGSPTGEALGRIGE

WLVTFYLLTDARLQGGGPGRRAAPAPIAT

>EST32145.1_*Streptomyces_niveus NCIMB 11891*

MTCVEEEVLARQLPAVPFYCPVEPAMHPGVDVLNDLTVDWMLRQNLDTDEHQRKRLAVCDFGGLTASTMP

YGRLEPLTLMAKIHAVLFSLDDGVCDETDATADLLAQETSRILRAVEAPAANSRSDSPHTAALRALRTEL

EQYASPQQVRRWTDAMRVYTSGLVWEASWRRSAELPSLNDYITLWMRAIGMAPTTAMIEVVGGFSVTDEE

LADPRVQALTEITWTLVSWDNDLYSRNKELLRAGDDLNLIDVLCHELGCEPREALNHAVAMRDRVMVLHG

RLSEQVLADASPELRSYLVGLGQFVRGHLDWASVCPRYSVPSGPAAQPGGWWKRHPSDAGREPLPIPTIS

WWWEQLAAPA

>KFE96946.1_*Chryseobacterium_luteum*

MNTPELLNEQSTYTDFATEQEPLQFYLRKFKDKQEYKCYPLYNEQVSKELIEWATKKSGIKKGTKAYQHT

ISSDISYWLALSFPLTKDLNKFKKTFYFFQMFSTMDDHADEDWGDGKADTEKIVKLWKKAIKLVDTIRDG

APWHIKALRTILMRMPNIPIYMRRNYSTMRKIMNDLSPVQRERYINSFKSYMENAMLQAQMSGKEKSTTL

QQYKEYRVKSIASIPCTLMVEYLYDIVLTNEEYFHPKLQELEKLGTLHVAFINDLFSLFKEYKGTFKNLH

HAVSIFILNEGLTFQQAIDKLCDEIEQLQKDYIDLKDEWFASGEYISDNVRRFIEGQQYYMAGNEKWHRL

SKRYHGENFNTTITSGTLKWSPEGTIYTPDDV

>KIZ18902.1_*Streptomyces_natalensis*

MPPVYAADSAPSRIGWELPPFYCPFNASLIHPKAEELEARAVEWIDRFGLYPDPTERAWGLATRGADFTS

RIIPYGDVEPMVLFAEWNYWAFALDDWQDAQDAGPARASAAAVADEGIRLFRSLEAPGSALLPPGPFTDA

LADLVQRTRALLTPYQLRRFGEGVRDWVLGATWQTANTERDVMPSLNDFCAMRMSVNGTRFTLTWCETAN

GAELPPDVLYAEPVQALTDAAGFVVSCDNDLFSYNKEDHQEPWEQNLVNVLVRERGCTPREAVRHAVALR

DQVLALVVRLGDKLARGANAPLRRYVEMLGEWIAGDVAYHALAPRYASPRNRNPLPVADASYDLVWADAP

SSARTAPPPVPSIAWWWAAERA

>ELQ82238.1_*Streptomyces_rimosus*

MAVRATVVIPQLYCPIPAAPHHETAGIERRVVEWMARFGFCDNDFHHAQVLANRTAEWACRIAPDGSSRL

LQIGADWSCLGLLLDDVYLDGGLFSRHPERFLPMAVQVIHGADHPETTDGGKADPYTVAFGEVSYRYRRH

ATGTVVRRWVDGVAEWFLAACVGMGQRASGTVPSLEEYFVIGPRDRGTKASIAVIEMAEGTSLPSEESET

PRIRALTQVASALVTFANDVYSYHREVKEQSLESNLVGILEHELRVPPQEAMTRAAALHDRLMCLYLALR

ERIARRATPEVRCYLGQLDHFIRGNLDYSAVSPRYRDDPGAAPDPAPAFGVWADSPSDGSLEPLPLRTVA

WWWDQLSPAA

>CFM47198.1_*Burkholderia_pseudomallei*

MPATPAPAPMPPARRLVGEACRIASIERSFERRFDPHRRALHAHCVRWIDEQSLAPAGSPPADLRALRYP

DLVAGYYVGAPPAVLEAIAGLSVWFFVWDDRHDEDARRLRRAAWARLRDALCAVLASPRARVADAEPIVG

ALSDCIVLRIRAWLGDAWNRRFTGHLRQMIDAYDDEFRARLAARIPTRDAYMRLRERTFGCEVWLDCLEL

AAGRALPDAVRAAPPYRLAGPARRSNSPRFTTISVRCARSAKPKKSTISAFR

>Q9X839.3_*Streptomyces_coelicolor* A3 (2)

MTQQPFQLPHFYLPHPARLNPHLDEARAHSTTWAREMGMLEGSGVWEQSDLEAHDYGLLCAYTHPDCDGP

ALSLITDWYVWVFFFDDHFLEKYKRSQDRLAGKAHLDRLPLFMPLDDAAGMPEPRNPVEAGLADLWTRTV

PAMSADWRRRFAVATEHLLNESMWELSNINEGRVANPVEYIEMRRKVGGAPWSAGLVEYATAEVPAAVAG

TRPLRVLMETFSDAVHLRNDLFSYQREVEDEGELSNGVLVLETFFGCTTQEAADLVNDVLTSRLHQFEHT

AFTEVPAVALEKGLTPLEVAAVGAYTKGLQDWQSGGHEWHMRSSRYMNKGERPLAGWQALTGPGTSAADV

GALLADAVAQRARSYTYVPFQKVGPSVIPDIRMPYPLELSPALDGARRHLSEWCREMGILSEGVWDEDKL

ESCDLPLCAAGLDPDATQDQLDLASGWLAFGTYGDDYYPLVYGHRRDLAAARLTTTRLSDCMPLDGEPVP

PPGNAMERSLIDLWVRTTAGMTPEERRPLKKAVDDMTEAWLWELSNQIQNRVPDPVDYLEMRRATFGSDL

TLGLCRAGHGPAVPPEVYRSGPVRSLENAAIDYACLLNDVFSYQKEIEYEGEIHNAVLVVQNFFGVDYPA

ALGVVQDLMNQRMRQFEHVVAHELPVVYDDFQLSEEARTVMRGYVTDLQNWMAGILNWHRNVPRYKAEYL

AGRTHGFLPDRIPAPPVPRSSPALTH

>AHH94051.1

MAMQPFELPTFYMPHPARLNPHLEGARVHTKAWAREFGMIEGSNVWDERQFDGMDYALLCAYTHPECDQEMLDLI

TDWYVWVFFFDDHFLEVFKRTKDQPGAKAYLDRLSLFMPHSGPITEKPTNQVEAGLEDLWNRTIPRMSDAWRQRF

IVSTENLLKDCVWELANIVEGRVANPIEYIEMRRKVGGAPWSADLVEVATEAEVPASVAHSRPLRVLKETFSDGV

HLRNDLFSYQREVEDEGENANAVLVLKEFLGYDTQRAADAVNDMITSRLHQFENTFFTELPLLCEENGLDPAERM

RVLVYARGLQDWQSGGHEWHMRSNRYMNEGGTAATASPSLGSFLGGPRGLGTATASIGSLVGLGG
