# Supplementary figures and images for: Exploring novel bacterial terpene synthases
Source: PLoS One. 2020 Apr 30;15(4):e0232220. doi: 10.1371/journal.pone.0232220 (PMC7192455; doi:10.1371/journal.pone.0232220)

**
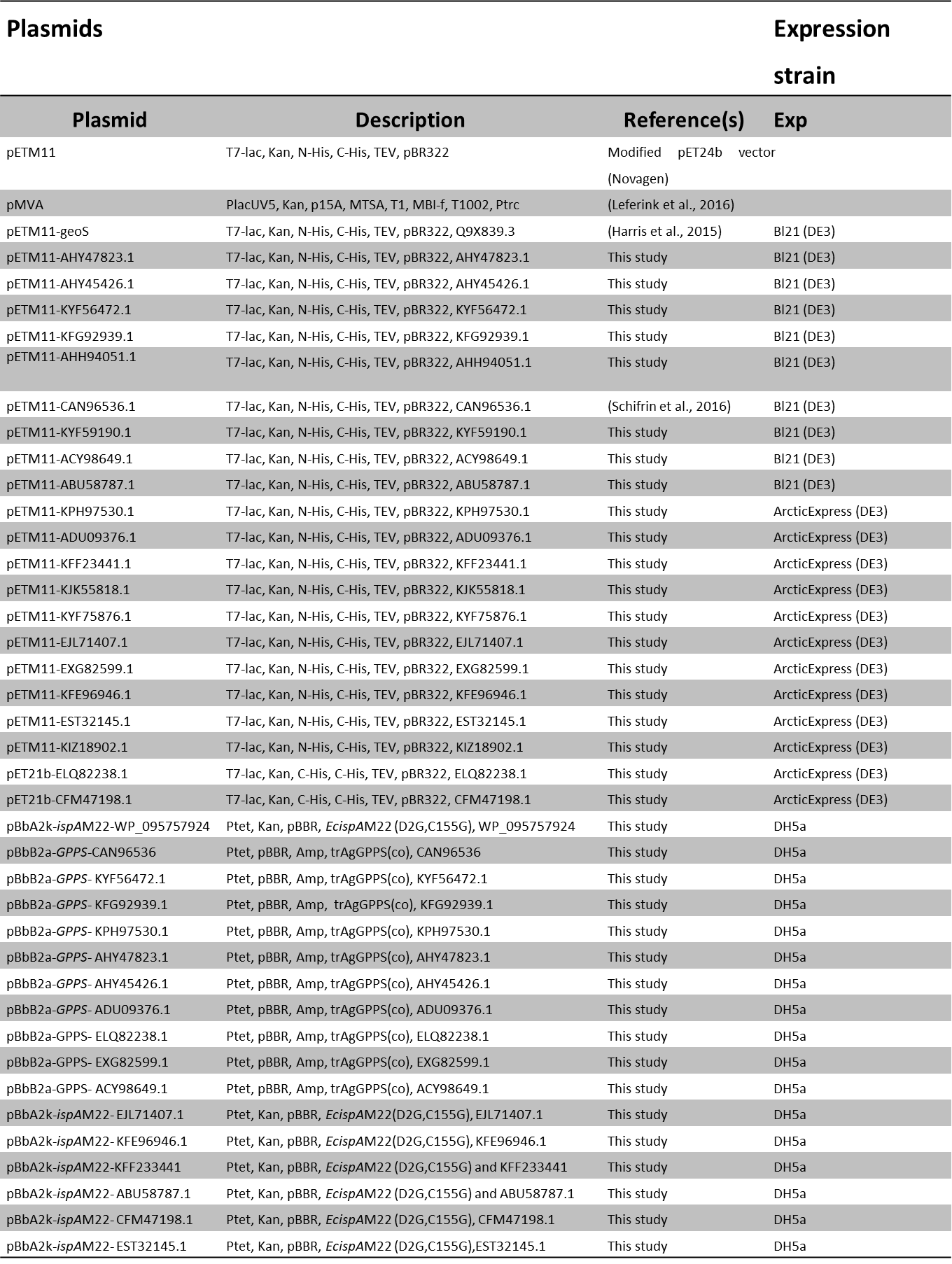
**

**S3 Table:** Plasmids used in this study

Supplement: S3 Table — (DOCX) [file pone.0232220.s003.docx]
