# Supplementary material for: Exploring novel bacterial terpene synthases
Source: PLoS One. 2020 Apr 30;15(4):e0232220. doi: 10.1371/journal.pone.0232220 (PMC7192455; doi:10.1371/journal.pone.0232220)
Supplement: S4 Table — Known TSs annotated in the tree in Fig 1 where the branches were indicated in colour based on their functionality: monoterpenoids (light blue), 2- methyisoborneol (orange), sesquiterpenoids (purple) and diterpenoids (blue). The same colour scheme is adapted here. (DOCX) [file pone.0232220.s004.docx]

|  | Enzyme | GenBank ID | NCBI-Protein ID | Organism |
| --- | --- | --- | --- | --- |
| 1 | 2-methylisoborneol synthase | WP_011333305 | ABA73584.1 | *Pseudomonas fluorescens PfO-1* |
| 2 |  | WP_047016550 | AKH83234.1 | *Streptomyces sp. CNQ-509* |
| 3 |  | WP_078984193 | AJT62038.1 | *Streptomyces lydicus* |
| 4 |  | GZL_09219 | AJC61740.1 | *Streptomyces sp. 769* |
| 5 |  | WP_011874125 | CAM02994.1 | *Saccharopolyspora erythraea NRRL 2338* |
| 6 |  | WP_037726550 | KFG06748.1 | *Streptomyces scabiei* |
| 7 |  | WP_039628838 | AJC52998.1 | *Streptomyces sp. 769* |
| 8 |  | WP_038524797 | AIA08360.1 | *Streptomyces albulus* |
| 9 |  | WP_020930496 | EXU89361.1 | *Streptomyces albulus PD-1* |
| 10 |  | WP_020938197 | AGS67713.1 | *Streptomyces collinus* |
| 11 |  | WP_046924697 | AJT62687.1 | *Streptomyces lydicus* |
| 12 |  | WP_012378420 | BAG18098.1 | *Streptomyces griseus subsp. griseus NBRC 13350* |
| 13 |  | WP_053126184 | AKZ53458.1 | *Streptomyces ambofaciens ATCC 23877* |
| 14 |  | WP_063481016 | ANB04309 1 | *Streptomyces ambofaciens* |
| 15 |  | WP_064537133 | ANH95154.1 | *Streptomyces sp. SAT1* |
| 16 |  | WP_011031839 | CAD55534.1 | *Streptomyces coelicolor A3(2)* |
| 17 |  | WP_041132328 | AJF68405.1 | *Streptomyces vietnamensis* |
| 18 |  | WP_015576150 | AGJ53442.1 | *Streptomyces sp. PAMC 26508* |
| 19 |  | WP_014157663 | ADW07061.1 | *Streptomyces pratensis ATCC 33331* |
| 20 |  | WP_062750724 | KYG53160.1 | *Streptomyces sp. WAC04657* |
| 21 |  | WP_055645320 | ALO06172.1 | *Streptomyces venezuelae* |
| 22 |  | WP_078095811 | AKL64808.1 | *Streptomyces sp. Mg1* |
| 23 |  | WP_014058647 | AEM85161.1 | *Streptomyces violaceusniger Tu 4113* |
| 24 | (4S,7S)-germacrene - D-4-ol synthase | WP_061940513 | AMP04969.1 | *Collimonas pratensis* |
| 25 | (+)-isodauc-8-en-11-ol synthase | WP_015031758 | CCA53839.1 | *Streptomyces venezuelae ATCC 10712* |
| 26 | (+)-intermedeol synthase | WP_003955204 | EDY49701.1 | *Streptomyces clavuligerus ATCC 27064* |
| 27 | (−)-neomeranol B synthase | WP_013004899 | CBG74359.1 | *Streptomyces scabei 87.22* |
|  |  |  |  |  |
| 28 | Geosmin synthase | WP_013004899 | KFG07762.1 | *Streptomyces scabiei (strain 87.22)* |
| 29 |  | YP_001612078 | CAN91958.1 | *Sorangium cellulosum So ce56* |
| 30 |  | YP_001866236 | ACC81293.1 | *Nostoc punctiforme PCC73102* |
| 31 |  | WP_013374506 | ADO68918.1 | *Stigmatella aurantiaca* |
| 32 |  | WP_014174668 | ADI05189.1 | *Streptomyces bingchenggensis BCW-1* |
| 33 |  | WP_075986266 | ALM38351.1 | *Streptomyces sp. FR-008* |
| 34 |  | WP_003951048 | EFE84188.2 | *Streptomyces albus J1074* |
| 35 |  | WP_040246537 | AJE81150.1 | *Streptomyces albus* |
| 36 |  | WP_014143690 | AEW95315.1 | *Streptomyces cattleya NRRL 8057* |
| 37 |  | WP_053562493 | ALC31536.1 | *Streptomyces sp. CFMR 7* |
| 38 |  | WP_015606689 | AGK75303.1 | *Streptomyces fulvissimus DSM 40593* |
| 39 |  | WP_058953825 | ALU92063.1 | *Streptomyces globisporus C-1027* |
| 40 |  | WP_012382258 | BAG23668.1 | *Streptomyces griseus subsp. griseus JCM 4626* |
| 41 |  | WP_044385074 | AJP04398.1 | *Streptomyces cyaneogriseus subsp. noncyanogenus* |
| 42 |  | WP_043504863 | AIS01158.1 | *Streptomyces glaucescens* |
| 43 |  | WP_012999852 | CBG69128.1 | *Streptomyces scabiei (strain 87.22)* |
| 44 |  | WP_010983603 | BAC69874.1 | *Streptomyces avermitilis MA-4680* |
| 45 |  | WP_053138925 | AKZ58833.1 | *Streptomyces ambofaciens ATCC 23877* |
| 46 |  | WP_063483426 | ANB09241.1 | *Streptomyces ambofaciens* |
| 47 |  | WP_061441960 | AIV33612.1 | *Streptomyces sp. CCM_MD2014* |
| 48 |  | WP_003972847 | AIJ12569.1 | *Streptomyces lividans TK24* |
| 49 |  | WP_064535845 | ANH94075.1 | *Streptomyces sp. SAT1* |
| 50 |  | WP_020942918 | AGS72508.1 | *Streptomyces collinus Tu 365* |
| 51 |  | WP_059253583 | CUW31946.1 | *Streptomyces reticuli* |
| 52 |  | WP_058082416 | ALO96950.1 | *Streptomyces hygroscopicus subsp. limoneus* |
| 53 |  | WP_014675700 | AEY92415.1 | *Streptomyces hygroscopicus subsp. jinggangensis* |
| 54 | Hedycaryol synthase | WP_014133196 | BAJ25873.1 | *Kitasatospora setae KM-6054* |
| 55 | Aristolochene synthase | WP_029184626 | GAQ51813 1 | *Streptomyces acidiscabies* |
| 56 | African-1-ene/African-2-ene synthase | SCLAV_p0985 | EFG04472.1 | *Streptomyces clavuligerus ATCC 27064* |
| 57 | (+)-caryolan-1-ol synthase | SGR_2079 | BAG18908.1 | *Streptomyces griseus subsp. griseus JCM 4626* |
| 58 | Clavulatriene A synthase | SSCG_05303 | EDY52275.1 | *Streptomyces clavuligerus ATCC 27064* |
| 59 | (+)-(2S,3S,9R)-pristinol synthase | WP_005320742 | EDY62784.1 | *Streptomyces pristinaespiralis* |
| 60 | Isoafricanol synthase | Strvi_5748 | AEM85259.1 | *Streptomyces violaceusniger* |
| 61 | Isohirsut-1-ene synthase | WP_003952619 | EDY47223.1 | *Streptomyces clavuligerus ATCC 27064* |
| 62 | Cucumene Synthase | SCLAV_p1407 | EFG04889.2 | *Streptomyces clavuligerus ATCC 27064* |
| 63 | 7-epi-α -eudesmol synthase | WP_003994861 | EFL36708.1 | *Streptomyces viridochromogenes DSM 40736* |
| 64 | R-linalool synthase | WP_003957954 | EDY52263.1 | *Streptomyces clavuligerus ATCC 27064* |
| 65 | (*E*)-β-caryophyllene synthase | WP_041318180 | CCH32724.1 | *Saccharothrix espanaensis DSM 44229* |
| 66 | Pentalenene synthase | WP_010984429 | BAC70709.1 | *Streptomyces avermitilis ATCC 31267* |
| 67 | 1,8-Cineole Synthase | WP_003952918 | EDY47508.1 | *Streptomyces clavuligerus ATCC 27065* |
| 68 | Avermitilol synthase | WP_010981512 | BAC67785.1 | *Streptomyces avermitilis MA-4680* |
| 69 | Allohedycaryol, (+)- synthase | WP_012394883 | ACC41646.1 | *Mycobacterium marinum ATCC BAA-535* |
| 70 | (-)-δ-cadinene synthase | WP_003954606 | EDY49122.1 | *Streptomyces clavuligerusATCC 27074* |
| 71 | (-)-δ-cadinene synthase | WP_003954606 | EFG03819.2 | *Streptomyces clavuligerus strain ATCC 27064* |
| 72 | (+)-Eremophilene synthase | WP_012241161 | CAN98722.1 | *Sorangium cellulosum So ce56* |
| 73 | α-Selinene synthase | WP_012190525 | ABX05626.1 | *Herpetosiphon aurantiacus DSM 785* |
| 74 | Obscuronatin synthase | Haur_2987 | ABX05625.1 | *Herpetosiphon aurantiacus* |
| 75 | (+)-t-muurolol synthase | WP_012119179 | ABU56748.1 | *Roseiflexus castenholzii DSM 13941* |
| 76 | (+)-t-muurolol synthase | WP_011958209 | ABQ91867.1 | *Roseiflexus sp. RS-1* |
| 77 | γ-cadinene synthase | WP_012792334 | ACU62166.1 | *Chitinophaga pinensis DSM 2588* |
| 78 | Cembrene C synthase | WP_011563485 | ABG03467.1 | *Rubrobacter xylanophilus DSM 9941* |
| 79 | Germacrene A synthase | WP_010998816 | BAB76384.1 | *Nostoc sp. PCC7120* |
| 80 | Germacrene A synthase | WP_011318775 | ABA21604.1 | *Anabaena variabilis ATCC 29413* |
| 81 | 8-epi-α-selinene synthase | WP_012410187 | ACC82216.1 | *Nostoc punctiforme PCC73102* |
| 82 | (+)-epicubenol synthase | SGR_6065 | BAG22894.1 | *Streptomyces griseus subsp. griseus JCM 4626* |
| 83 | Tsukubadiene Synthase | stsu_20912 | EIF90392.1 | *Streptomyces tsukubaensis NRRL 18488* |
| 84 | (+)-4-epi-cubebol synthase | WP_043653400 | ACZ89572.1 | *Streptosporangium roseum DSM 43021* |
| 85 | β-himachalene synthase | WP_035852539 | EXG82620.1 | *Cryptosporangium arvum DSM 44712* |
| 86 | Sodorifen synthase | WP_004943913 | AGO55049.1 | *Serratia plymuthica 4Rx13* |
| 87 | Cattleyene synthase | WP_014150548 | AEW99846.1 | *Streptomyces cattleya* |
| 88 | (+)-(1(10)E,4E,6S,7R)-germacradien-6-ol synthase | YP_004922572 | ADW03055.1 | *Streptomyces pratensis aTCC 33331* |
| 89 | Selina-4(15),7(11)-diene synthase | WP_005317515 | EDY64907.2 | *Streptomyces pristinaespiralis ATCC 25486* |
| 90 | Selina-4(15),7(11)-diene synthase | WP_003982050 | ELQ82161.1 | *Streptomyces rimosus ATCC 10970* |
| 91 | Epi-isozizaene synthase | WP_030765460 | AMM08369.1 | *Streptomyces albidoflavus* |
| 92 |  | WP_107071290 | AJE82448.1 | *Streptomyces albus BK3-25* |
| 93 |  | WP_099052949 | AIS00442.1 | *Streptomyces glaucescens* |
| 94 |  | S.col_Tü365 | AGS71733.1 | *Streptomyces collinus Tü365* |
| 95 |  | S.hyg_limKCTC1717 | ALO96063.1 | *Streptomyces hygroscopicus subsp. limoneus KCTC 1717* |
| 96 |  | WP_044388300 | AJP05813.1 | *Streptomyces cyaneogriseus subsp. noncyanogenus* |
| 97 |  | WP_047122496 | CQR61979.1 | *Streptomyces leeuwenhoekii* |
| 98 |  | WP_058917971 | ALV54576.1 | *Streptomyces sp. 4F* |
| 99 |  | WP_107083301 | BAC70743.1 | *Streptomyces avermitilis MA-4680* |
| 100 |  | WP_107416269 | ALV33657.1 | *Streptomyces sp. CdTB01* |
| 101 |  | WP_063483044 | ANB08470.1 | *Streptomyces ambofaciens* |
| 102 |  | S.liv_TK24 | AIJ13444.1 | *Streptomyces lividans TK24* |
| 103 |  | WP_008415715 | EFE83901.1 | *Streptomyces albus J1074* |
| 104 |  | WP_011030119 | CAB94607.1 | *Streptomyces coelicolor A3(2)* |
| 105 | Corvol ether B synthase | WP_014134444 | BAJ27126.1 | *Kitasatospora setae KM-6054* |
| 106 | Hydropyrene Synthase | WP_003963279 | EFG04252.2 | *Streptomyces clavuligerus ATCC 27064* |
| 107 | (+)-t-muurolol synthase | WP_003956090 | EDY50541.1 | *Streptomyces clavuligerus ATCC 27064* |
| 108 | (+)-t-muurolol synthase | WP_003956090 | EFG03561.1 | *Streptomyces clavuligerus ATCC 27074* |
| 109 | Labda-7,13(16),14-triene synthase | WP_003954347 | EFG03981.1 | *Streptomyces clavuligerus ATCC 27064* |
| 110 | Isopimara-8,15-diene synthase | WP_012181499 | ABV97190.1 | *Salinispora arenicola CNS-205* |

**S4 table: List of terpene synthases annotated in phylogenetic tree in Fig. 1**

Known TSs annotated in the tree in Fig 1 where the branches were indicated in colour based on their functionality: monoterpenoids (light blue), 2- methyisoborneol (orange), sesquiterpenoids (purple) and diterpenoids (blue). The same colour scheme is adapted here.
