# Supplementary material for: Exploring novel bacterial terpene synthases
Source: PLoS One. 2020 Apr 30;15(4):e0232220. doi: 10.1371/journal.pone.0232220 (PMC7192455; doi:10.1371/journal.pone.0232220)
Supplement: S1 Fig — Neighbour-joining tree constructed from the amino acid sequences of characterized terpene synthases in this study (underlined) and in previous studies. Geosmin synthases (dark red), 2-methylisoborneol synthases (orange), monoterpene (light blue), diterpene (dark blue) and sesquiterpene synthases (purple) are shown and uncharacterized bacterial terpene synthases in this study (in red). (DOCX) [file pone.0232220.s005.docx]

**
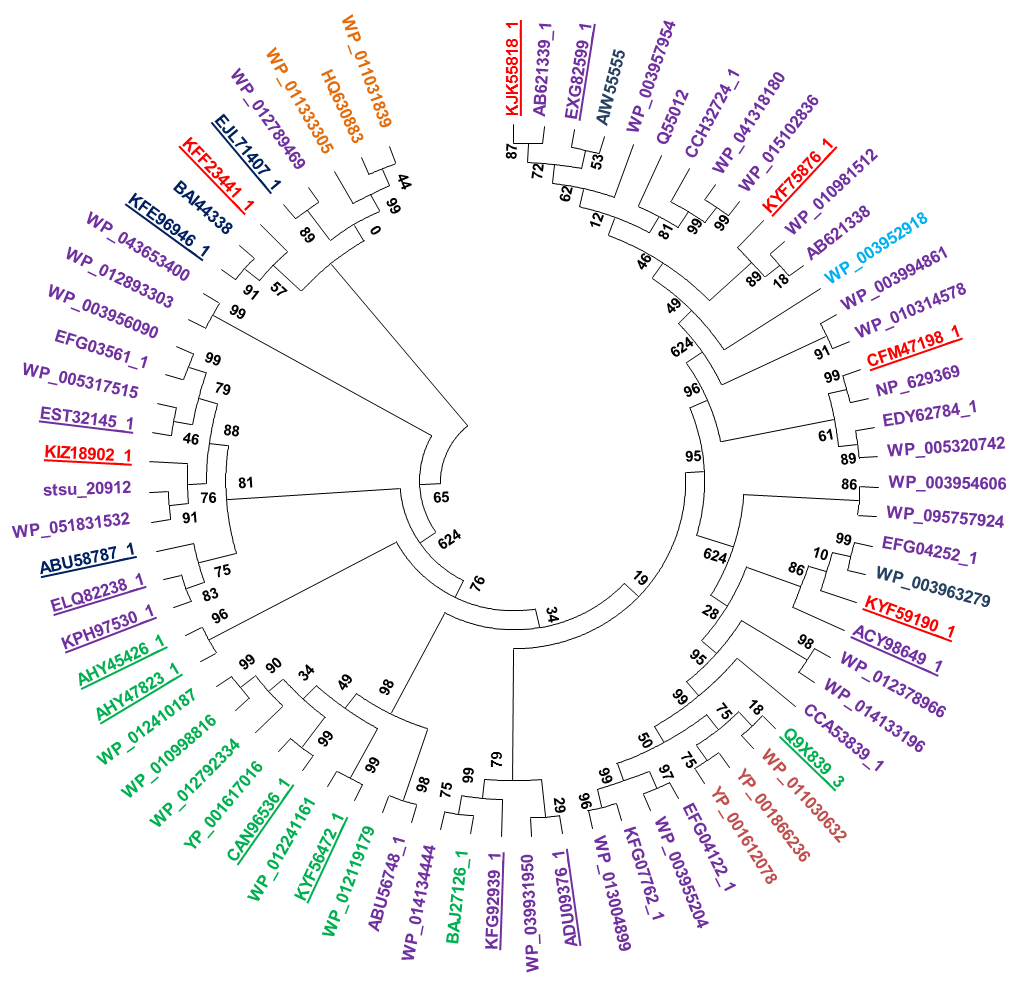
**

**S1 Fig**: **Bacterial terpene synthases neighbour-joining tree**

Neighbour-joining tree constructed from the amino acid sequences of characterized terpene synthases in this study (underlined) and in previous studies. Geosmin synthases (dark red), 2-methylisoborneol synthases (orange), monoterpene (light blue), diterpene (dark blue) and sesquiterpene synthases (purple) are shown and uncharacterized bacterial terpene synthases in this study (in red).
