# Supplementary material for: Exploring novel bacterial terpene synthases
Source: PLoS One. 2020 Apr 30;15(4):e0232220. doi: 10.1371/journal.pone.0232220 (PMC7192455; doi:10.1371/journal.pone.0232220)
Supplement: S2 Fig — GC-MS traces showing the separation of geosmin (0.1 mg mL-1) on a HP5 column and its retention time at 7.5 minutes. B. Mass spectra of geosmin. (DOCX) [file pone.0232220.s006.docx]

**
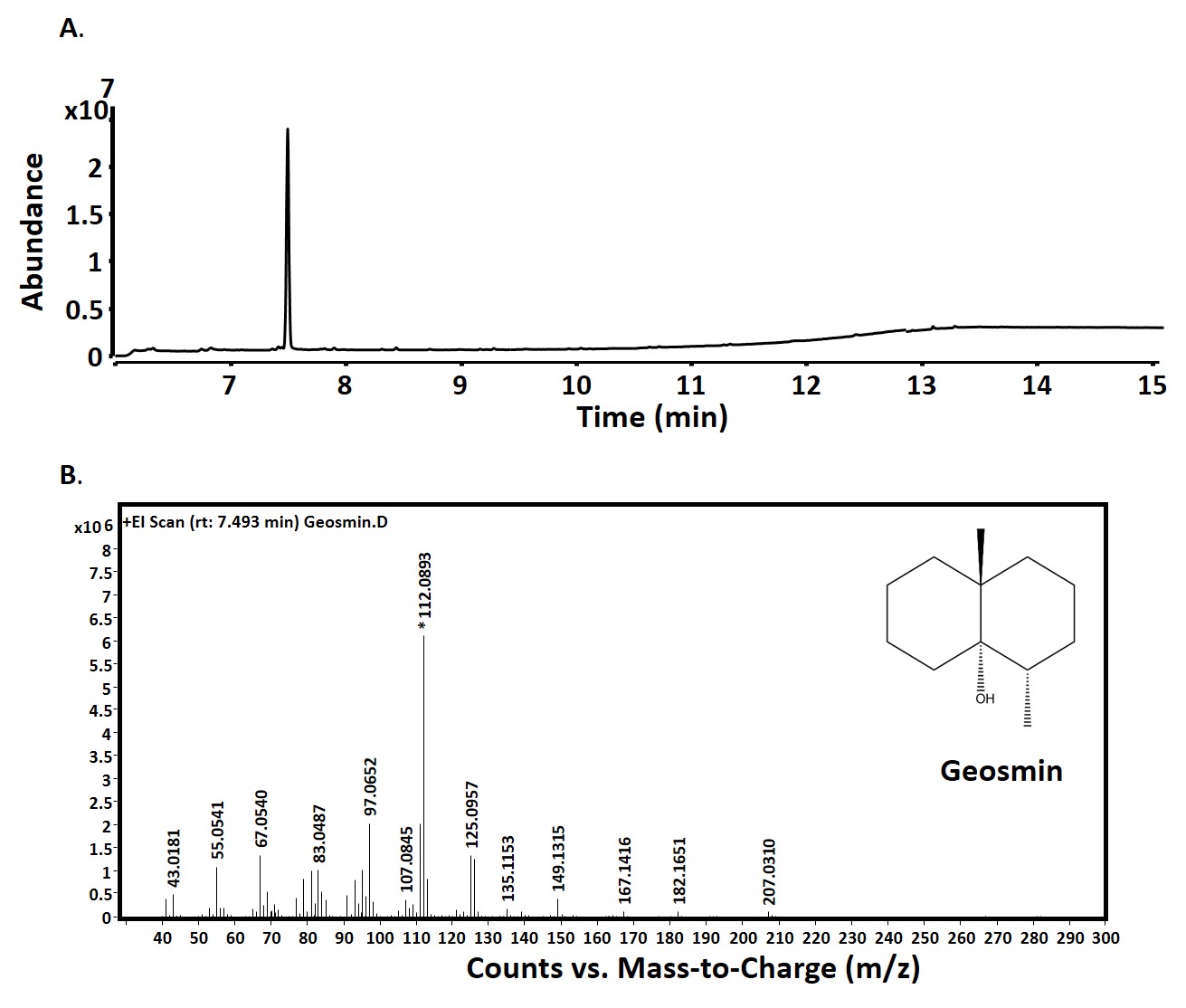
**

**S2 Fig: GC-MS traces of geosmin standard**.

GC-MS traces showing the separation of geosmin (0.1 mg mL^-1^) on a HP5 column and its retention time at 7.5 minutes. B. Mass spectra of geosmin.
