# Supplementary material for: Exploring novel bacterial terpene synthases
Source: PLoS One. 2020 Apr 30;15(4):e0232220. doi: 10.1371/journal.pone.0232220 (PMC7192455; doi:10.1371/journal.pone.0232220)
Supplement: S3 Fig — GC-MS chromatogram of geosmin synthase with 75 μM of FPP. Peak 1: geosmin (rt: 7.5), peak 2: Germacrene D (rt: 7.86), peak 3: germacradienol (rt: 8.84). B. Mass spectra of compounds observed in the extracts. (DOCX) [file pone.0232220.s007.docx]

**
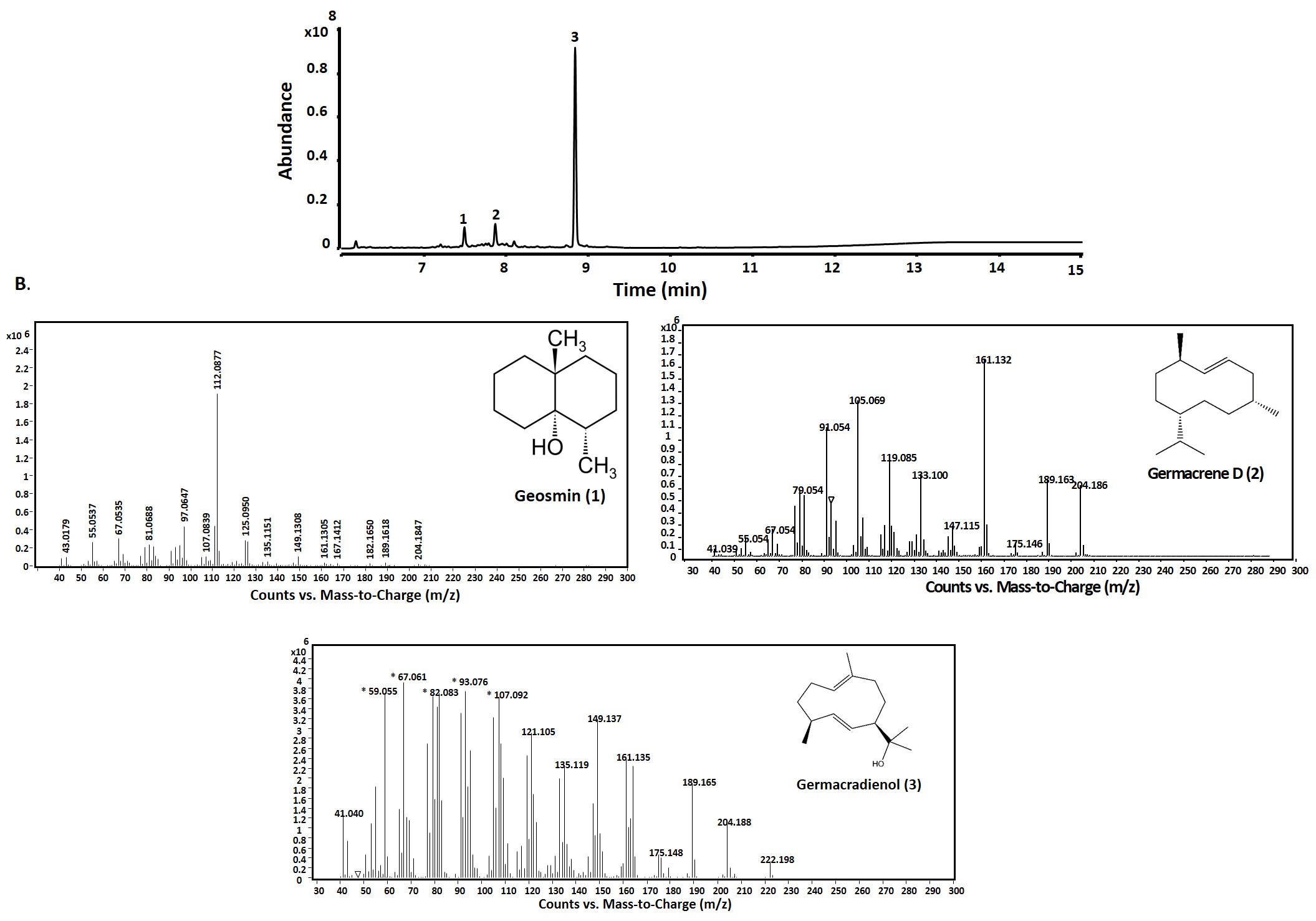
**

**S3 Fig: GC-QToF analysis of n-hexane extracts obtained from *in vitro* assays**

GC-MS chromatogram of geosmin synthase with 75 µM of FPP. Peak 1: geosmin (rt: 7.5), peak 2: Germacrene D (rt: 7.86), peak 3: germacradienol (rt: 8.84). B. Mass spectra of compounds observed in the extracts.
