# Supplementary material for: Exploring novel bacterial terpene synthases
Source: PLoS One. 2020 Apr 30;15(4):e0232220. doi: 10.1371/journal.pone.0232220 (PMC7192455; doi:10.1371/journal.pone.0232220)
Supplement: S4 Fig — A. GC-MS traces showing the separation of limonene (0.1 mg mL-1) produced in this study on a HP5 column. The internal standard, sec-butyl benzene (0.1%, v/v), has a retention time of 9.5 minutes. Peak 1: R- Limonene (rt: 9.41). B: Mass spectra of limonene. (DOCX) [file pone.0232220.s008.docx]

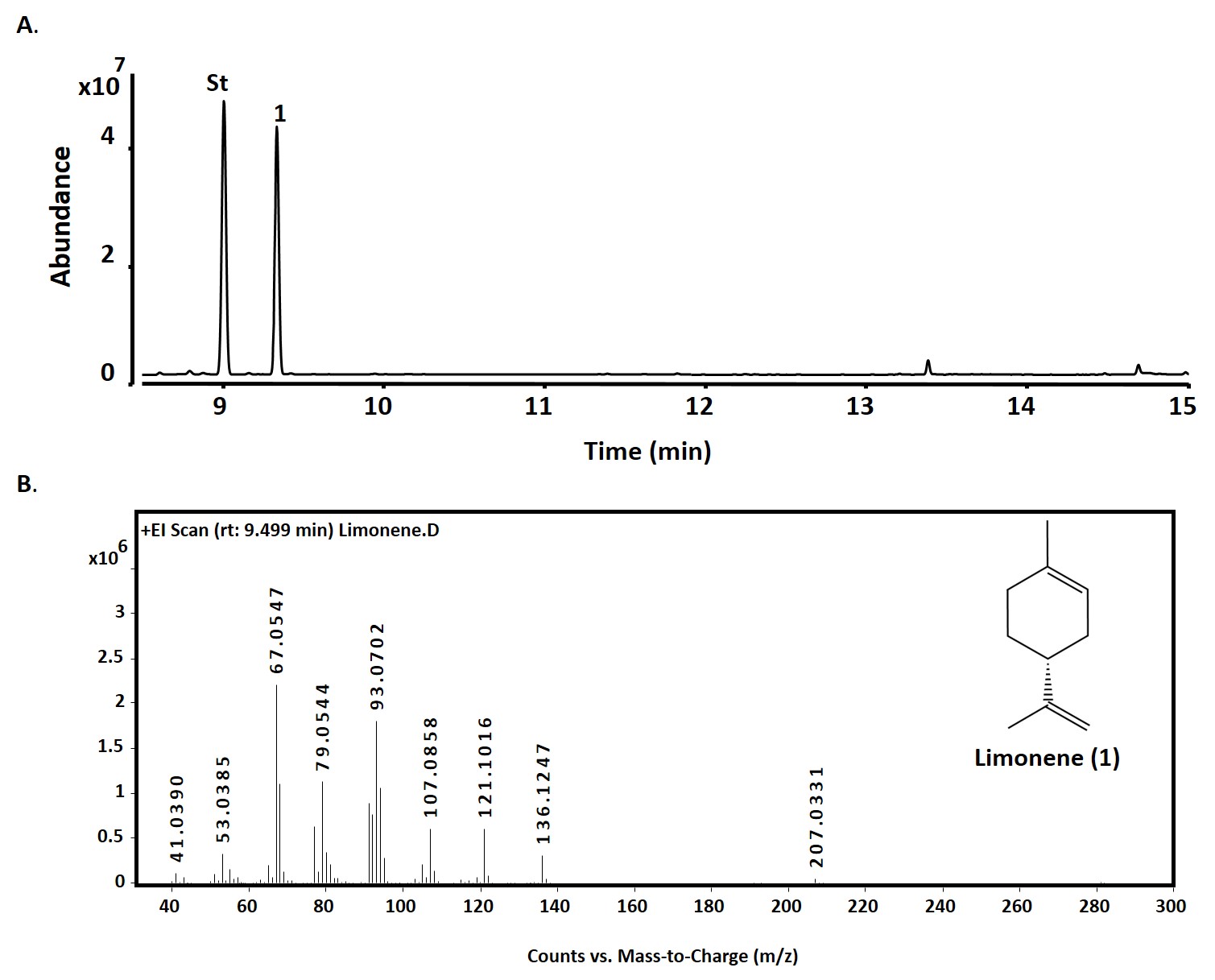


**S4 Fig: GC-QToF analysis of limonene standard**

A. GC-MS traces showing the separation of limonene (0.1 mg mL^-1^) produced in this study on a HP5 column. The internal standard, sec-butyl benzene (0.1%, v/v), has a retention time of 9.5 minutes. Peak 1: R- Limonene (rt: 9.41). B: Mass spectra of limonene.
