# Supplementary material for: Exploring novel bacterial terpene synthases
Source: PLoS One. 2020 Apr 30;15(4):e0232220. doi: 10.1371/journal.pone.0232220 (PMC7192455; doi:10.1371/journal.pone.0232220)
Supplement: S5 Fig — A. GC chromatogram of 10 μg of limonene synthase (pJBEI-6410) crude extracts with 75 μM of GPP. Peak 1: limonene (rt: 9.41). B. Mass spectra of limonene. (DOCX) [file pone.0232220.s009.docx]

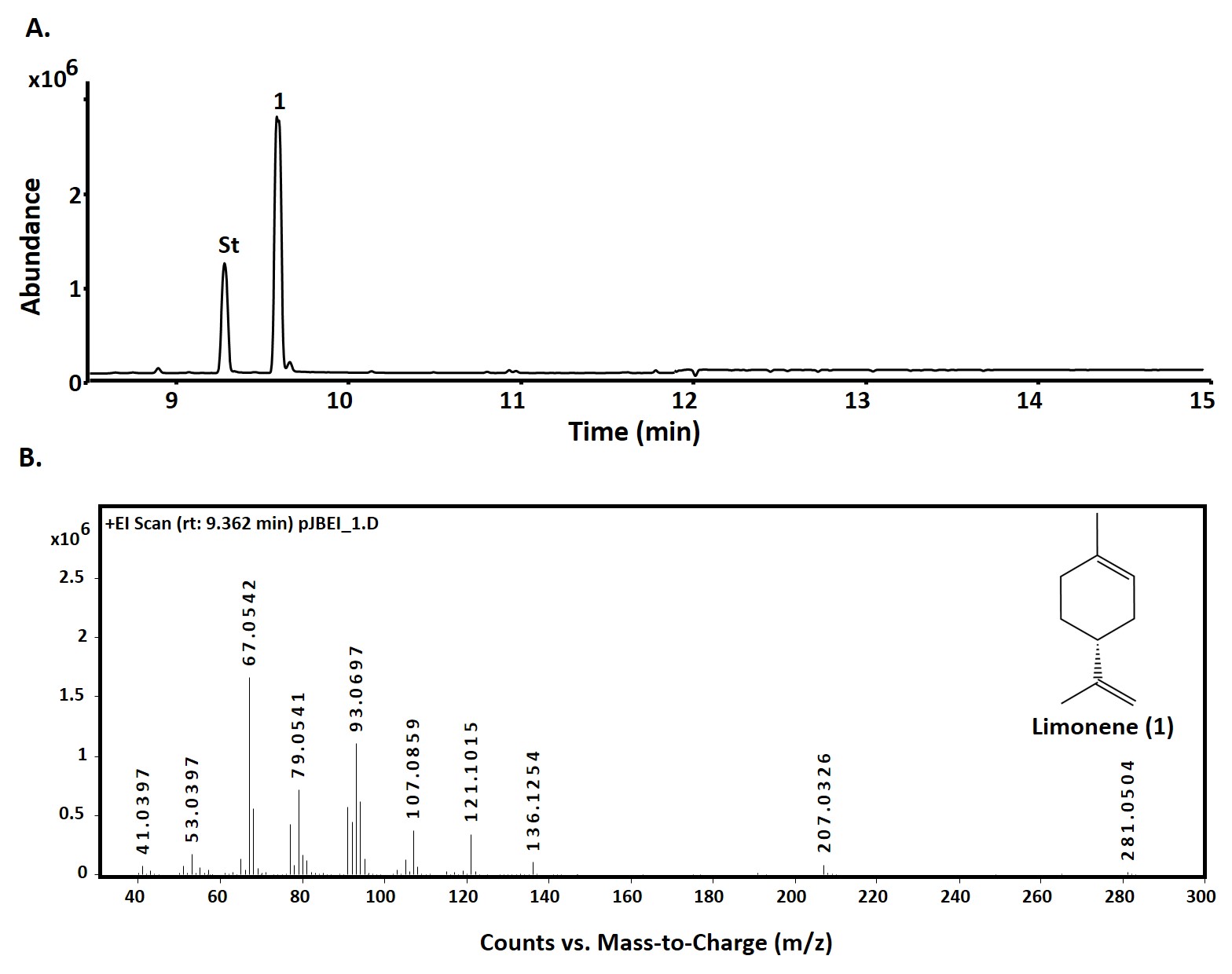


**S5 Fig: GC-QToF analysis of nonane extracts obtained from limonene synthase *in vitro* assays**

**A.** GC chromatogram of 10 µg of limonene synthase (pJBEI-6410) crude extracts with 75 µM of GPP. Peak 1: limonene (rt: 9.41). B. Mass spectra of limonene.
