# Supplementary material for: Exploring novel bacterial terpene synthases
Source: PLoS One. 2020 Apr 30;15(4):e0232220. doi: 10.1371/journal.pone.0232220 (PMC7192455; doi:10.1371/journal.pone.0232220)
Supplement: S6 Fig — A. GC-MS traces showing the separation of standard nerolidol mix (0.1 mg mL-1) on a HP5 column. B. MS spectra of -cis and -trans Nerolidol. C. GC-MS chromatogram of trans-nerolidol produced by AHY47823 with FPP. D. Mass spectra for trans-nerolidol produced by AHY47823. (DOCX) [file pone.0232220.s010.docx]

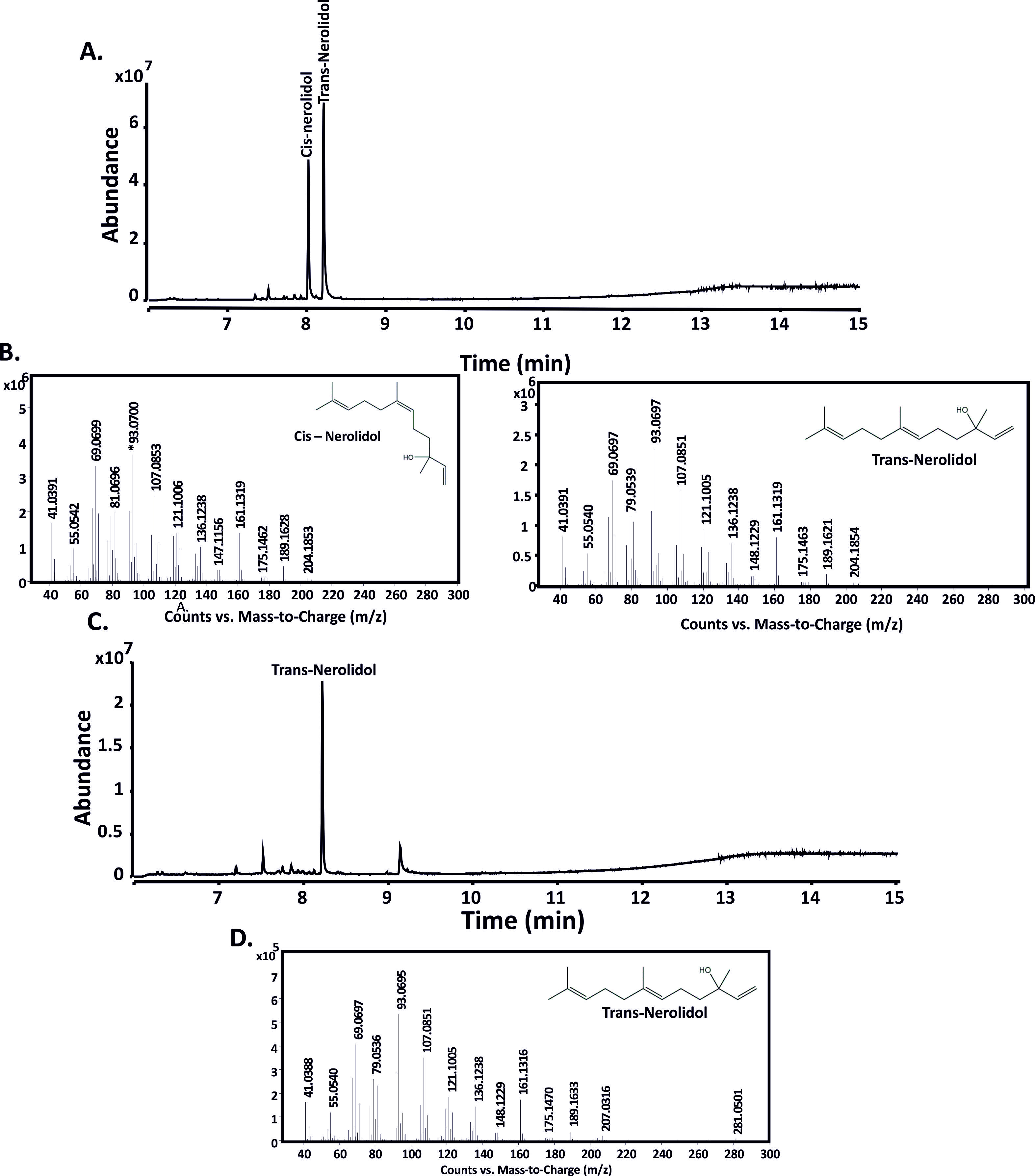


**S6 Fig: GC-QToF analysis of nerolidol standard mix and products obtained by AHY47823 upon incubation with FPP.** **A.** GC-MS traces showing the separation of standard nerolidol mix (0.1 mg mL^-1^) on a HP5 column. **B.** MS spectra of -cis and -trans Nerolidol. **C**. GC-MS chromatogram of trans-nerolidol produced by AHY47823 with FPP. **D.** Mass spectra for trans-nerolidol produced by AHY47823.
