# Supplementary material for: Exploring novel bacterial terpene synthases
Source: PLoS One. 2020 Apr 30;15(4):e0232220. doi: 10.1371/journal.pone.0232220 (PMC7192455; doi:10.1371/journal.pone.0232220)
Supplement: S7 Fig — A. GC-MS traces showing the separation of standard linalool mix (0.1 mg mL-1) on a CP-Chirasil-DEX-CB column. B. GC-MS chromatogram of linalool isomers produced by AHY47823 with FPP. Samples were analyzed by GC on an Agilent Technologies 7890A GC system equipped with an FID detector, a 7693 auto sampler, and a CP-Chirasil-DEX-CB column (25 m × 0.25 mm i.d., 0.25 μm film thickness). For linalool, the program initiated at a temperature of 70°C which was then increased to 90°C at 8°C/min. This was followed by an increase in temperature to 150°C at a rate of 2°C/min and then to 190°C at 40°C/min (1 min hold). The FID detector was maintained at a temperature of 200°C with a flow of hydrogen at 30 mL/min. (DOCX) [file pone.0232220.s011.docx]

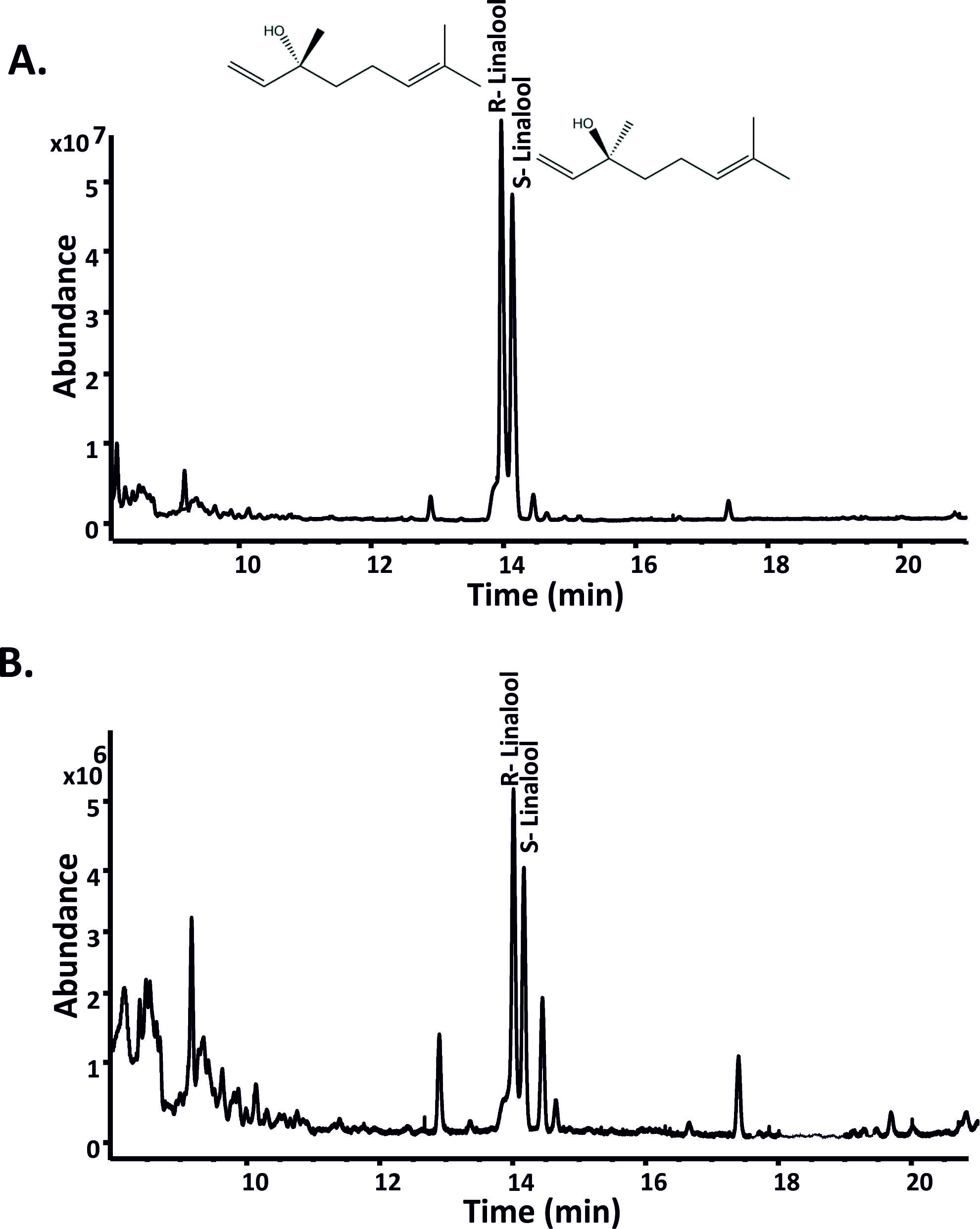


**S7 Fig: GC-QToF analysis of linalool standard mix and products obtained by AHY47823 upon incubation with GPP**. A. GC-MS traces showing the separation of standard linalool mix (0.1 mg mL^-1^) on a CP-Chirasil-DEX-CB column. **B.** GC-MS chromatogram of linalool isomers produced by AHY47823 with FPP. Samples were analyzed by GC on an Agilent Technologies 7890A GC system equipped with an FID detector, a 7693 auto sampler, and a CP-Chirasil-DEX-CB column (25 m × 0.25 mm i.d., 0.25 μm film thickness). For linalool, the program initiated at a temperature of 70 °C which was then increased to 90 °C at 8 °C/min. This was followed by an increase in temperature to 150 °C at a rate of 2 °C/min and then to 190 °C at 40 °C/min (1 min hold). The FID detector was maintained at a temperature of 200 °C with a flow of hydrogen at 30 mL/min.
