# Supplementary material for: Exploring novel bacterial terpene synthases
Source: PLoS One. 2020 Apr 30;15(4):e0232220. doi: 10.1371/journal.pone.0232220 (PMC7192455; doi:10.1371/journal.pone.0232220)
Supplement: S9 Fig — Total ion chromatograms of products obtained from an incubation RrNerS (left) and RrBerS (right) with FPP for 3 hours at variable temperatures. Samples were analyzed by GC-QToF on HP5 column. (DOCX) [file pone.0232220.s013.docx]

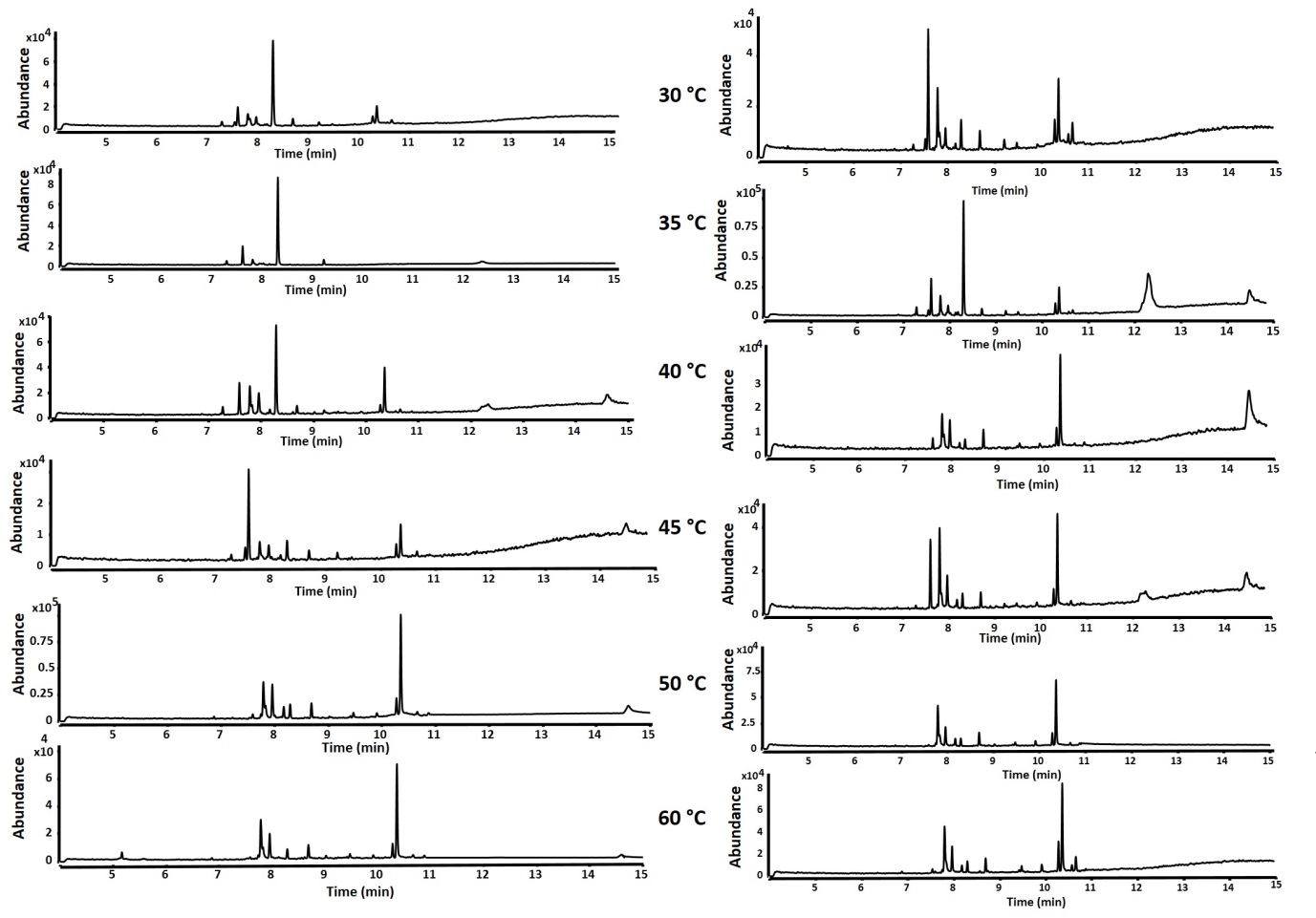


**S9 Fig:** Total ion chromatograms of products obtained from an incubation RrNerS (left) and RrBerS (right) with FPP for 3 hours at variable temperatures. Samples were analyzed by GC-QToF on HP5 column.
