# Supplementary material for: Exploring novel bacterial terpene synthases
Source: PLoS One. 2020 Apr 30;15(4):e0232220. doi: 10.1371/journal.pone.0232220 (PMC7192455; doi:10.1371/journal.pone.0232220)
Supplement: S10 Fig — The reference spectra from the NIST library shown in blue and the compound spectra shown in red.A. α–bergamotene produced by RrBerS, B. Aromandendrene produced by ScAroS, C and D. cadinene and longiborneol produced by BpLonS, E. germacrene D produced byKaGerS, F and G. copaene and acora-3,7(14)–diene produced byAbAcoS, and H and I. β-elemene and 1(10),4,7(11)-germacra-triene produced by MiGerS. (DOCX) [file pone.0232220.s014.docx]

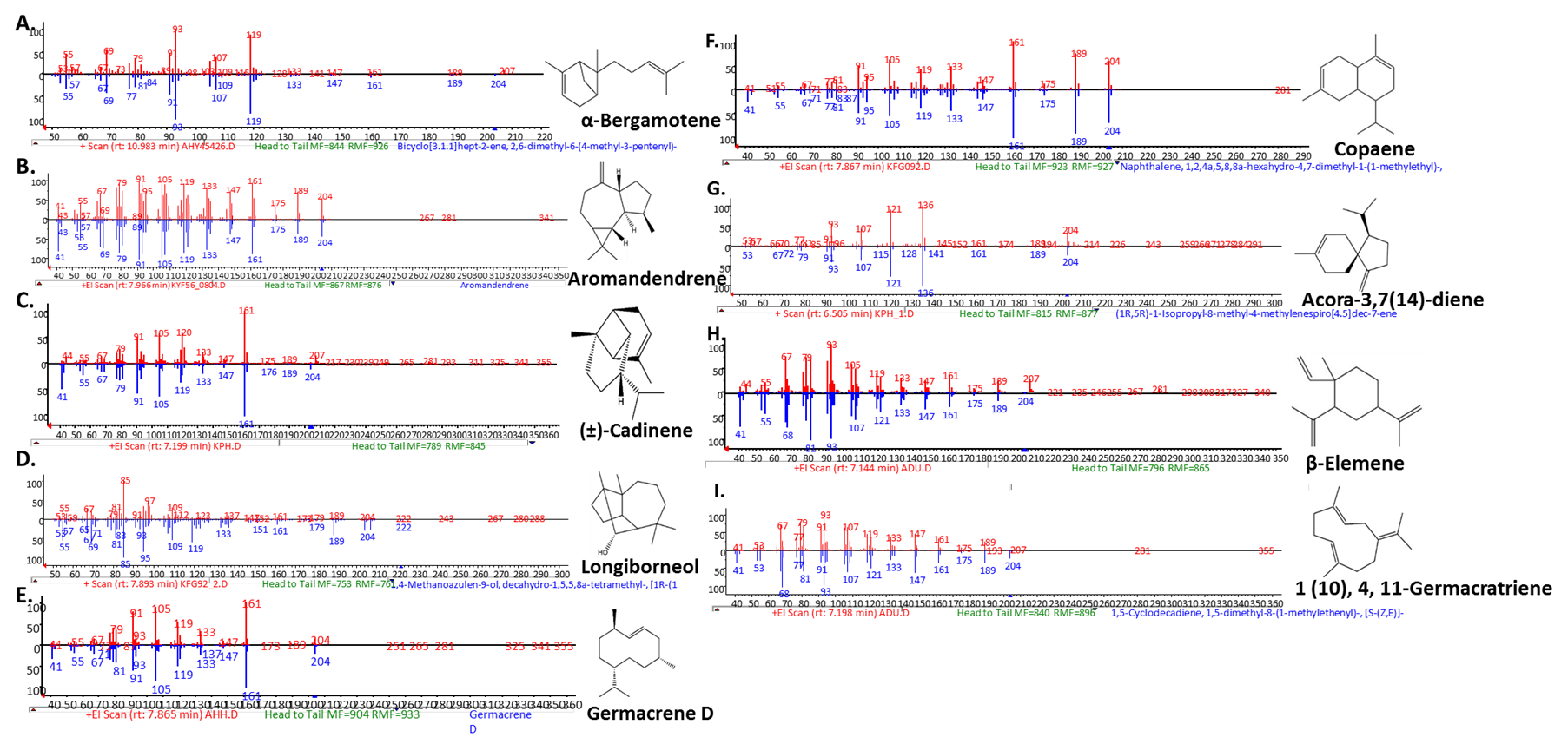


**S10 Fig**: **Comparison of obtained mass spectra with NIST Library spectra.** The reference spectra from the NIST library shown in blue and the compound spectra shown in red.**A.** α – bergamotene produced by RrBerS, **B.** Aromandendrene produced by ScAroS, **C and D**. cadinene and longiborneol produced by BpLonS, **E.** germacrene D produced byKaGerS , **F** and **G.** copaene and acora-3,7(14)–diene produced byAbAcoS , and **H** and **I.** β-elemene and 1(10),4,7(11)-germacra-triene produced by MiGerS.
