# Supplementary material for: Exploring novel bacterial terpene synthases
Source: PLoS One. 2020 Apr 30;15(4):e0232220. doi: 10.1371/journal.pone.0232220 (PMC7192455; doi:10.1371/journal.pone.0232220)
Supplement: S11 Fig — A. GC-MS traces showing the separation of monoterpenoids (0.1 mg mL-1) standards used in this study on a HP5 column. The internal standard used, sec-butyl benzene (St; 0.1% v/v), has a retention time of 9 minutes. Peak 1: cis-ocimene (rt: 9.56), peak 2: trans-ocimene (rt: 9.72), 3: linalool (rt: 10.43). B. Comparison of obtained mass spectra with NIST Library spectra and standard as shown in A. of products yielded by CAN96536 with GPP in in vitro assay. (DOCX) [file pone.0232220.s015.docx]

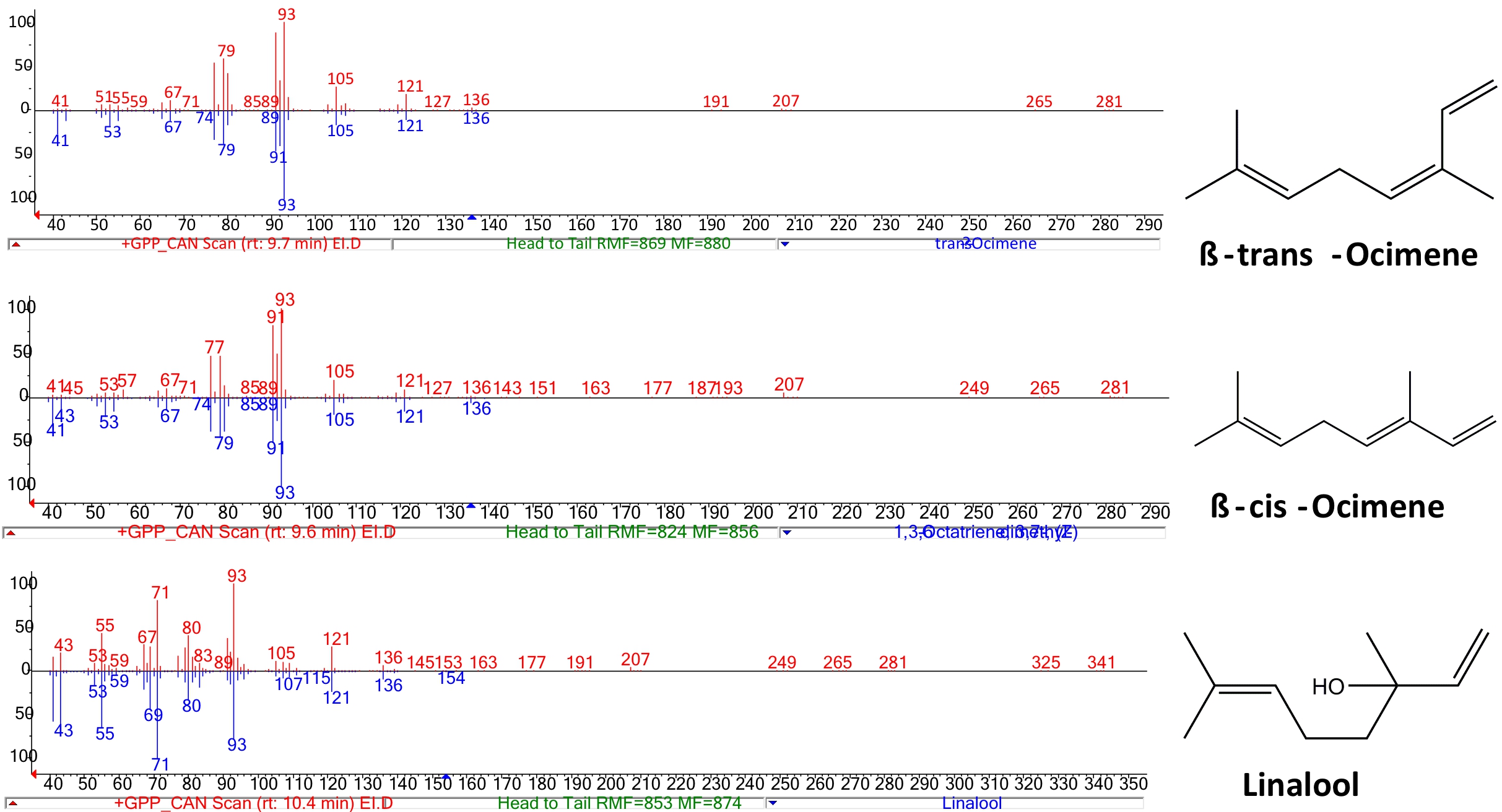


**S11 Fig: GC-MS traces of authentic monoterpenoid standards**.

1. GC-MS traces showing the separation of monoterpenoids (0.1 mg mL^-1^) standards used in this study on a HP5 column. The internal standard used, sec-butyl benzene (St; 0.1% v/v), has a retention time of 9 minutes. Peak 1: cis-ocimene (rt: 9.56), peak 2: trans-ocimene (rt: 9.72), 3: linalool (rt: 10.43). **B**. Comparison of obtained mass spectra with NIST Library spectra and standard as shown in A. of products yielded by CAN96536 with GPP in *in vitro* assay.
