# Supplementary material for: Exploring novel bacterial terpene synthases
Source: PLoS One. 2020 Apr 30;15(4):e0232220. doi: 10.1371/journal.pone.0232220 (PMC7192455; doi:10.1371/journal.pone.0232220)
Supplement: S12 Fig — GC-MS chromatograms of extracts profile with FPP are shown for A. BpTPS; B. ScTPS1; C. CvTPS; D. SsTPS; E. ScTPS2; F. SniTPS; G.SnaTPS. The peaks in the chromatogram do not correspond to any terpenoids. (DOCX) [file pone.0232220.s016.docx]

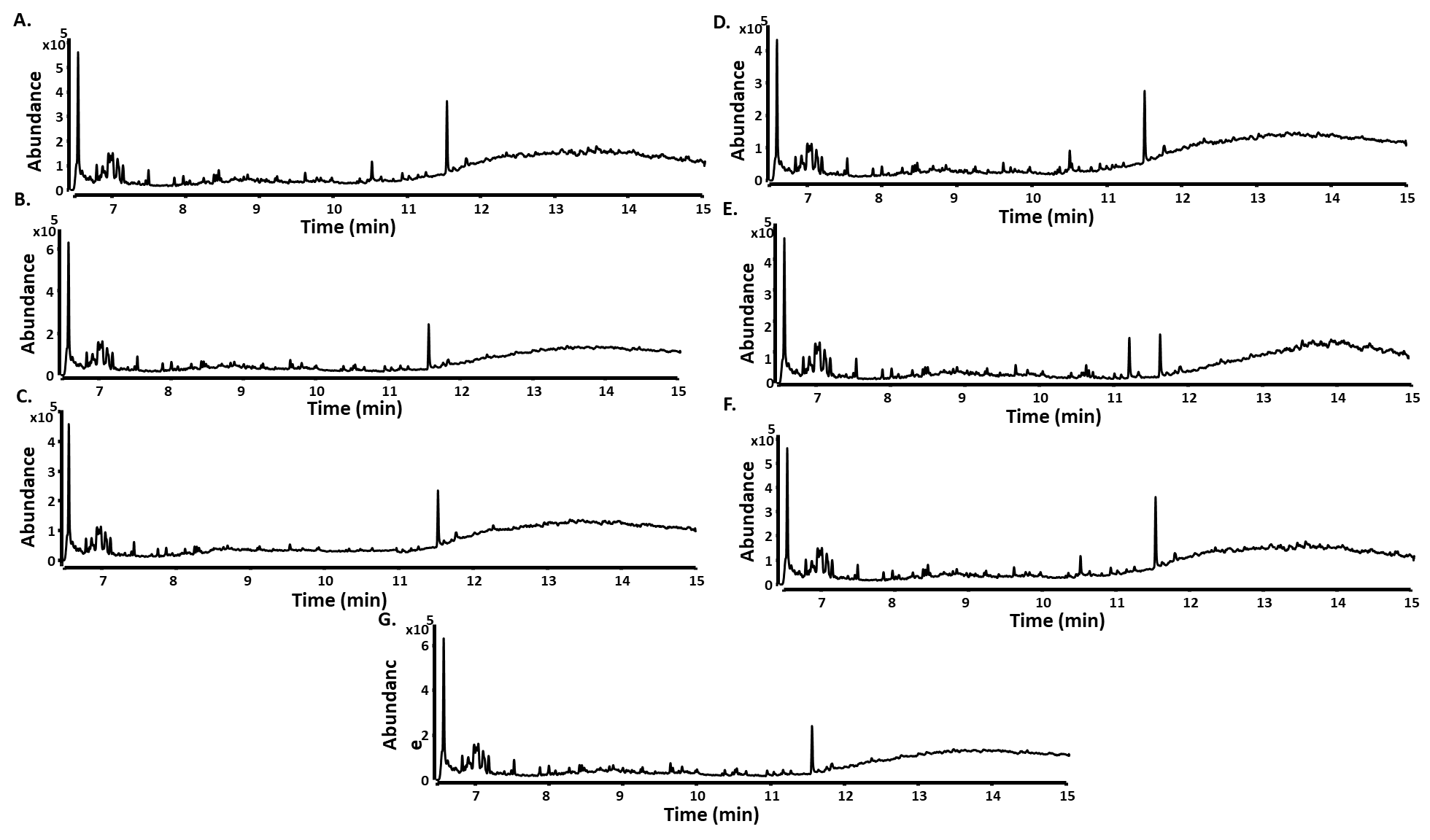


**S12 Fig**: **GC-QToF analysis of n-hexane extracts from *in vitro* assays obtained with selected TSs**.

GC-MS chromatograms of extracts profile with FPP are shown for **A.** BpTPS; **B.** ScTPS1; **C.** CvTPS; **D.** SsTPS; **E.** ScTPS2; **F.** SniTPS; **G**.SnaTPS. The peaks in the chromatogram do not correspond to any terpenoids.
