# Supplementary material for: Exploring novel bacterial terpene synthases
Source: PLoS One. 2020 Apr 30;15(4):e0232220. doi: 10.1371/journal.pone.0232220 (PMC7192455; doi:10.1371/journal.pone.0232220)
Supplement: S13 Fig — A. GC-MS chromatogram for geosmin synthase products, B. mass spectra for Germacradienol, C—G. Comparison of obtained mass spectra with NIST Library spectra of products yielded by GeoS. (DOCX) [file pone.0232220.s017.docx]

**
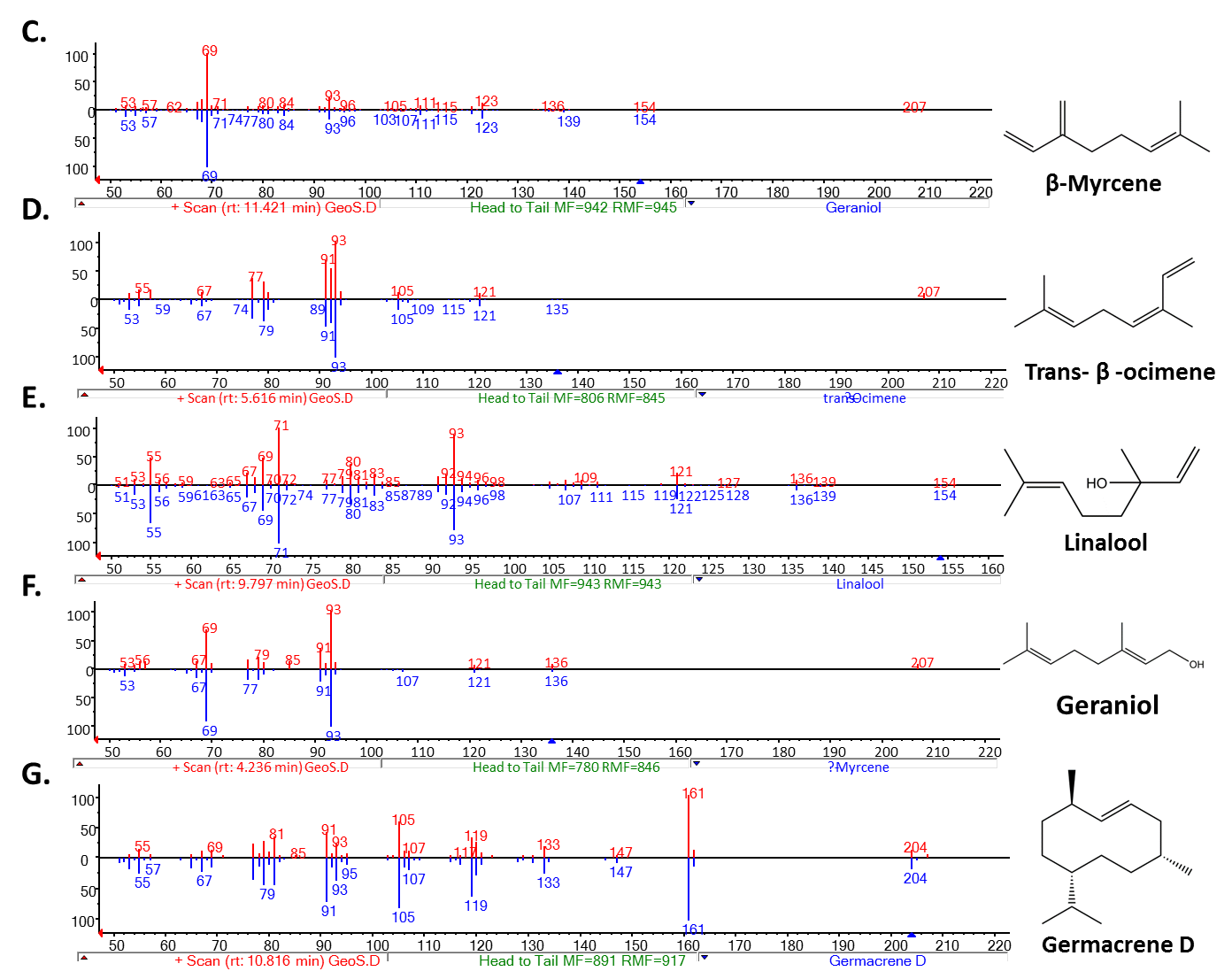
**

**S13 Fig: Comparison of obtained mass spectra with NIST Library spectra of products yielded by geosmin synthase in *in viv*o conditions**. **A.** GC-MS chromatogram for geosmin synthase products, **B.** mass spectra for Germacradienol, **C - G**. Comparison of obtained mass spectra with NIST Library spectra of products yielded by GeoS.
