# Supplementary material for: Exploring novel bacterial terpene synthases
Source: PLoS One. 2020 Apr 30;15(4):e0232220. doi: 10.1371/journal.pone.0232220 (PMC7192455; doi:10.1371/journal.pone.0232220)
Supplement: S14 Fig — A–B. SrGuaS C. TcCubS D. CaCubS. (DOCX) [file pone.0232220.s018.docx]

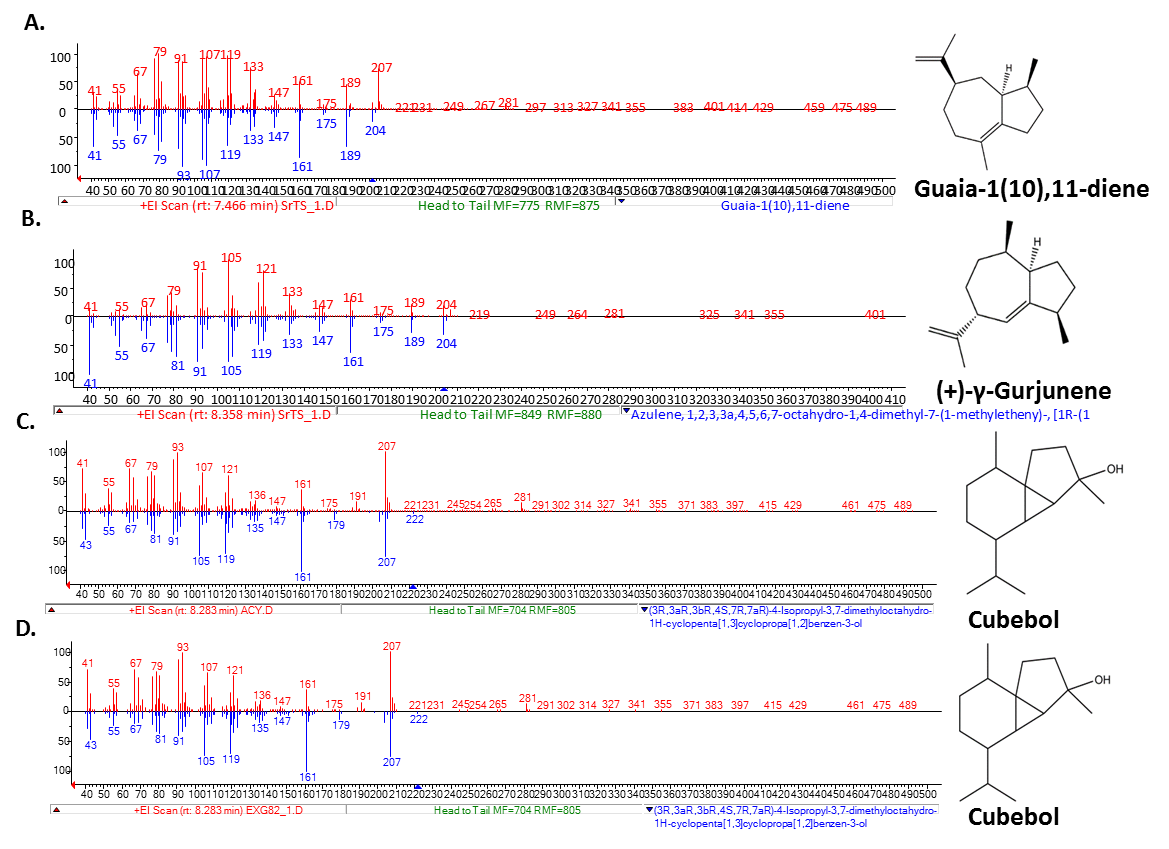


**S14 Fig:** Comparison of obtained mass spectra with NIST Library spectra of products yielded by TSs in *in vivo* conditions. **A – B.** SrGuaS **C.** TcCubS **D.** CaCubS.
