# Supplementary material for: Exploring novel bacterial terpene synthases
Source: PLoS One. 2020 Apr 30;15(4):e0232220. doi: 10.1371/journal.pone.0232220 (PMC7192455; doi:10.1371/journal.pone.0232220)
Supplement: S15 Fig — A. spata-13,17-diene produced by overexpression of ispAM22 and WP_095757924 in E. coli. B. Mass spectra of produced spata-13,17-diene. (DOCX) [file pone.0232220.s019.docx]

**
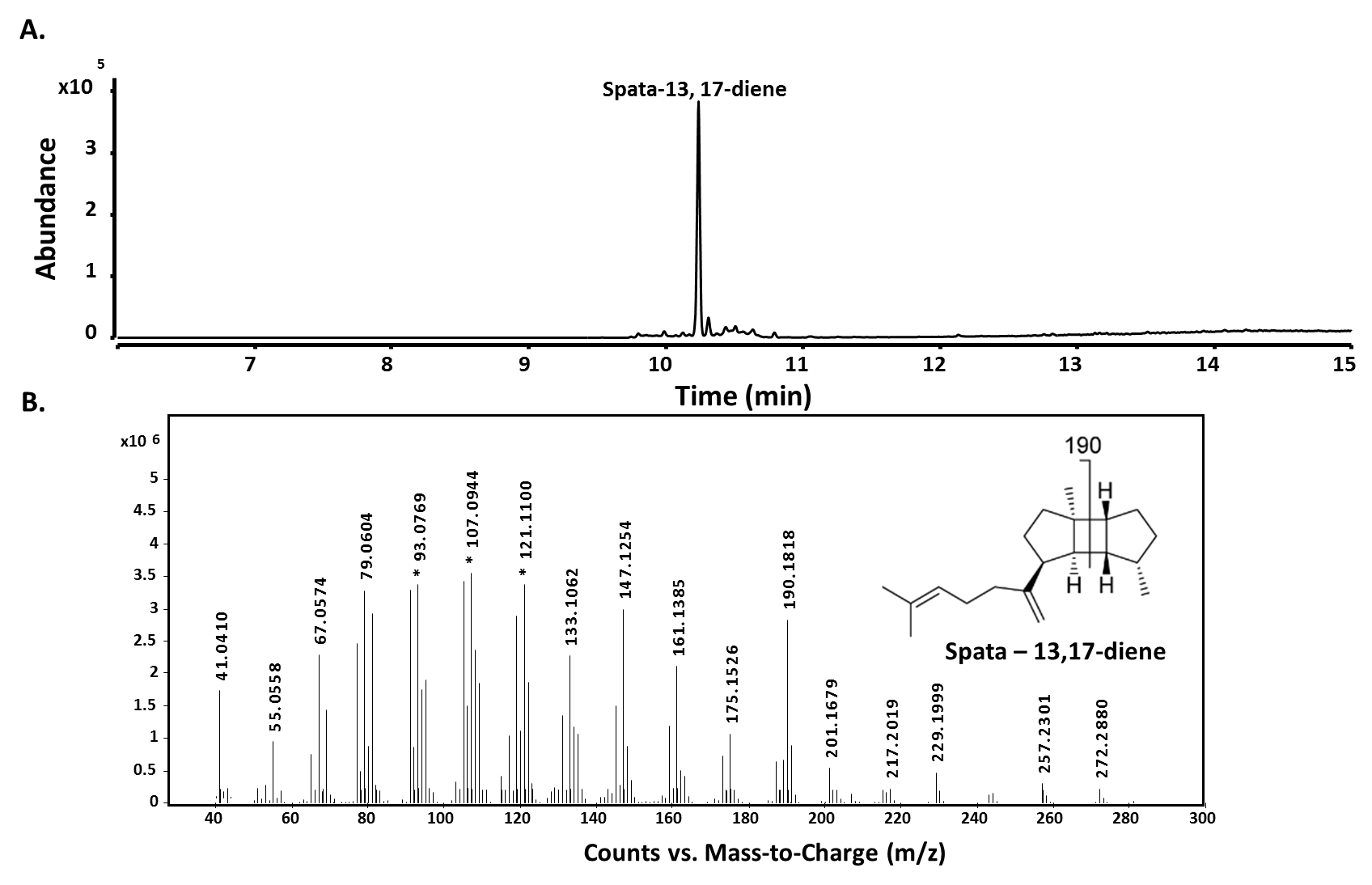
**

**S15 Fig: GC-QToF analysis of spata-13, 17-diene from nonane extracts of *in vivo* production in *E. coli*. A.** spata-13,17-diene produced by overexpression of ispAM22 and WP_095757924 in *E. coli.* B. Mass spectra of produced spata-13,17-diene.
