# Supplementary material for: Exploring novel bacterial terpene synthases
Source: PLoS One. 2020 Apr 30;15(4):e0232220. doi: 10.1371/journal.pone.0232220 (PMC7192455; doi:10.1371/journal.pone.0232220)
Supplement: S18 Fig — (DOCX) [file pone.0232220.s022.docx]

**
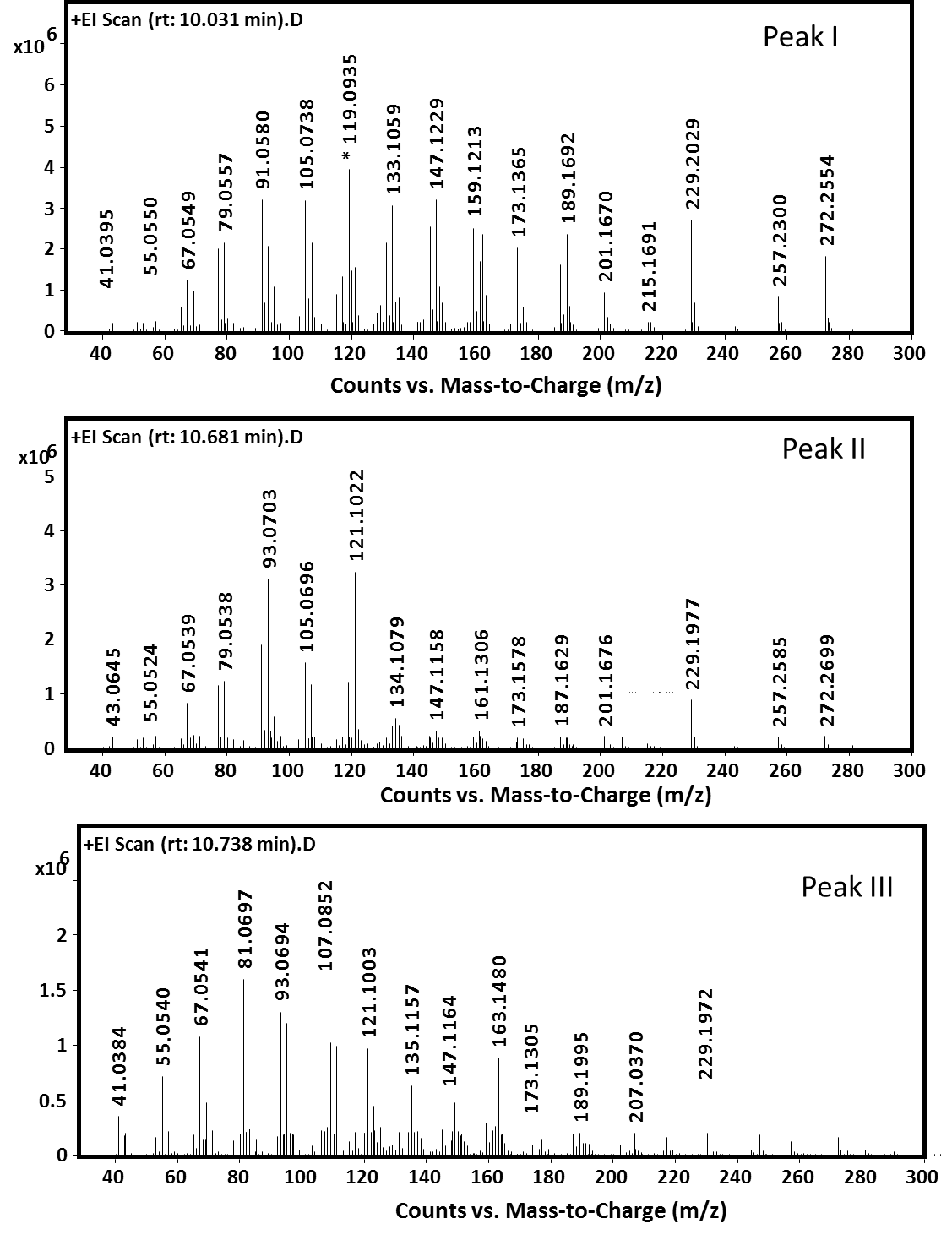
**

**S18 Fig:** Mass-spectra of compounds diterpenoid produced by ClDTPS corresponding to the peaks annotated in Fig. 5C.
